# BOFdat: Generating biomass objective functions for genome-scale metabolic models from experimental data

**DOI:** 10.1371/journal.pcbi.1006971

**Published:** 2019-04-22

**Authors:** Jean-Christophe Lachance, Colton J. Lloyd, Jonathan M. Monk, Laurence Yang, Anand V. Sastry, Yara Seif, Bernhard O. Palsson, Sébastien Rodrigue, Adam M. Feist, Zachary A. King, Pierre-Étienne Jacques

**Affiliations:** 1 Département de Biologie, Université de Sherbrooke, Sherbrooke, Québec, Canada; 2 Department of Bioengineering, University of California, San Diego, La Jolla, CA, United States of America; 3 Bioinformatics and Systems Biology Program, University of California, San Diego, La Jolla, CA, United States of America; 4 Department of Pediatrics, University of California, San Diego, La Jolla, CA, United States of America; 5 Novo Nordisk Foundation Center for Biosustainability, Technical University of Denmark, Kemitorvet, Lyngby, Denmark; Hebrew University of Jerusalem, ISRAEL

## Abstract

Genome-scale metabolic models (GEMs) are mathematically structured knowledge bases of metabolism that provide phenotypic predictions from genomic information. GEM-guided predictions of growth phenotypes rely on the accurate definition of a biomass objective function (BOF) that is designed to include key cellular biomass components such as the major macromolecules (DNA, RNA, proteins), lipids, coenzymes, inorganic ions and species-specific components. Despite its importance, no standardized computational platform is currently available to generate species-specific biomass objective functions in a data-driven, unbiased fashion. To fill this gap in the metabolic modeling software ecosystem, we implemented BOFdat, a Python package for the definition of a **B**iomass **O**bjective **F**unction from experimental **dat**a. BOFdat has a modular implementation that divides the BOF definition process into three independent modules defined here as steps: 1) the coefficients for major macromolecules are calculated, 2) coenzymes and inorganic ions are identified and their stoichiometric coefficients estimated, 3) the remaining species-specific metabolic biomass precursors are algorithmically extracted in an unbiased way from experimental data. We used BOFdat to reconstruct the BOF of the *Escherichia coli* model *i*ML1515, a gold standard in the field. The BOF generated by BOFdat resulted in the most concordant biomass composition, growth rate, and gene essentiality prediction accuracy when compared to other methods. Installation instructions for BOFdat are available in the documentation and the source code is available on GitHub (https://github.com/jclachance/BOFdat).

## Author summary

The formulation of phenotypic predictions by genome-scale models (GEMs) is dependent on the specified objective. The idea of a biomass objective function (BOF) is to represent all metabolites necessary for cells to double so that optimizing the BOF is equivalent to optimizing growth. Knowledge of the qualitative and quantitative organism’s composition (i.e. which metabolites are necessary for growth and in what proportion) is critical for accurate predictions. We implemented BOFdat with the idea that experimental data should drive the definition of the biomass composition. As omic datasets become more available, the possibility of integrating them to obtain a condition-specific biomass composition is in reach and therefore one of the main features of BOFdat. While major macromolecules, coenzymes, and inorganic ions are ubiquitous components across species, several species-specific components exist in the cell that should be accounted for in the BOF. To identify these, we implemented an approach that minimizes the error between experimental essentiality data and GEM-driven prediction. Hence BOFdat provides an unbiased, data-driven approach to defining BOF that has the potential to improve the quality of new genome-scale models and greatly decrease the time required to generate a new reconstruction.

This is a *PLOS Computational Biology* Software paper.

## Introduction

Genome-scale metabolic models (GEMs) are widely used to generate phenotypic predictions from genomic information, with wide-ranging applications from discovery to metabolic engineering [1]. To compute growth phenotypes, one must first define a biomass objective function (BOF) that encompasses all the major components of the cell, using data drawn from experimental measurements and literature [2,3]. The typical BOF primarily includes the cell’s major macromolecules (DNA, RNA, protein, lipids, carbohydrates), crucial coenzymes and inorganic ions, species-specific metabolites such as cell wall components and finally growth and non-growth associated maintenance costs which represent basal energy requirements to sustain the cell and divide. The formulation of a BOF parameterized with these elements allows the computation of phenotypes on different media, such as growth rate, nutrient requirements, and biosynthetic potential. Dikicioglu *et al*. studied the importance of the quantitative definition of the BOF and showed its impact on model behavior and predictions [4]. Xavier *et al*. attempted to organize the content of the BOF of existing genome-scale models to understand the phylogenetic relationship of cellular compositions in prokaryotes [5]. While their work yielded information on the qualitative composition of existing BOF, they also noted that no systematic computational framework exists for its definition.

Experimental measurements of biomass composition have been developed, and it is already known that the cellular composition varies upon different growth conditions. Ratios between DNA, RNA and proteins have been shown to vary depending on growth rate and nutrient availability [6]. The growth rate also affects cellular volume [7], which in turn impacts the total cell weight and the proportion of its components. Finally, Beck *et al*. recently reviewed and provided a state of the art method of determining experimental macromolecular composition, and showed that the macromolecular composition varies considerably from one species to another [8]. While these studies clearly showed diversity across species and conditions in terms of biomass composition and the impact of the BOF on model predictions, modelers often default to copy the BOF of a quality GEM rather than generating their own. The lack of a defined computational workflow facilitating the inclusion of experimental data to the BOF of GEMs may account for this behavior within the modeling community.

Previously developed tools provide modelers with either a biased or unbiased approach to define the BOF for a metabolic reconstruction. Biased approaches have the benefit of being based on current biological knowledge but are hampered by potential fluctuations in metabolic objectives over specific conditions or across species and strains. For example, the SEED platform [9] automates the reconstruction process and lets users decide on the objective function from a list of BOF defined according to phylogenetic groups, i.e. one BOF for all gram-negative bacteria. However, the unbiased computational extraction of metabolic objectives from experimental data is expected to better represent the content and objectives of the cell. Such approaches have been developed by other groups and include ObjFind [10], BOSS [11] and more recently invFBA [12]. These methods use fluxomics data to predict the metabolic end goals of the cell [13], an approach that is currently limited by the availability of this type of data, and by the small number of fluxes generated by fluxomics experiments. Here we present **BOFdat**, a Python software package for the complete definition of organism-specific BOF from experimental data. This package embeds a systematic 3-step procedure, where each step can be performed independently, that generates both qualitative and quantitative definitions of the BOF. BOFdat takes advantage of nowadays routinely generated multiple omic datasets (genomic, transcriptomic, proteomic and lipidomic) as well as the increasingly available gene essentiality data [14] to generate a BOF specific to the organism of interest.

## Methods

### A computational workflow for biomass definition from experimental data

**Fig 1:**
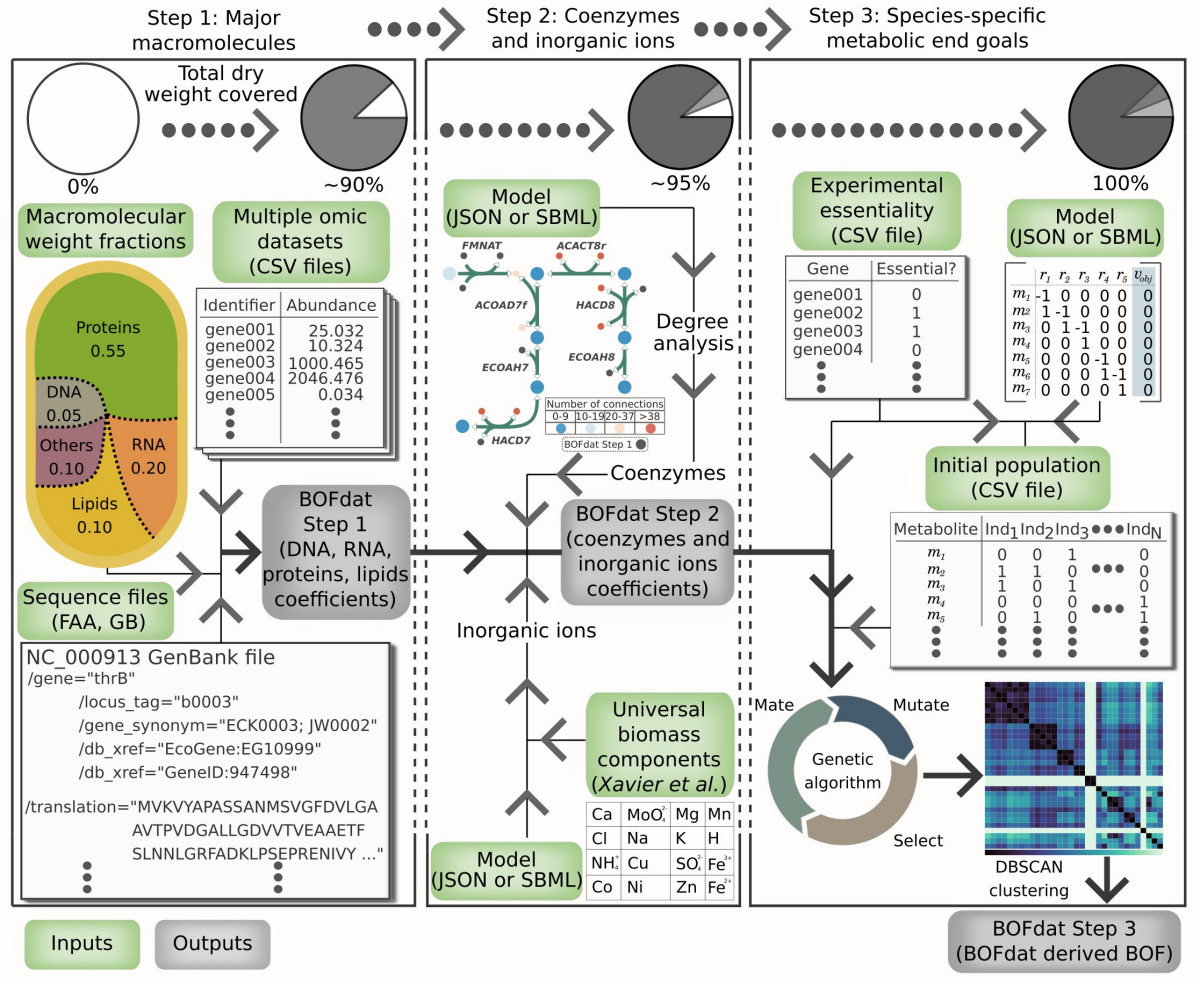
The three-step workflow for generating biomass objective functions from experimental data with BOFdat. Each step is presented in a rectangular frame in which input, and output files are shown using green or grey boxes, respectively. The modular implementation of BOFdat allows performing each step sequentially or independently, i.e. Step 3 can be used by itself to improve the gene essentiality prediction of an existing BOF. When the sequence of the workflow is observed, the output biomass function from Step 1 and 2 is the input for the subsequent step. Following the light arrows leads from the input to the output of each Step. The thicker arrows present the normal workflow for BOFdat leading to the final output of Step 3.

In a GEM, the BOF is represented as a reaction in the stoichiometric matrix, where metabolites are consumed or produced in a proportion given by each stoichiometric coefficient to represent the net requirements for generation of cell biomass at steady state [15]. Feist and Palsson [2] previously divided the definition of the BOF for a given organism into three different levels, based on the amount of knowledge available for the organism. A basic level BOF includes major macromolecules of the cell (DNA, RNA, proteins, and lipids), an intermediate BOF provides the polymerization and maintenance costs of macromolecules and the cell in general, whereas an advanced BOF definition includes metabolites that are specific to the organism of interest (coenzymes, inorganic ions, cell wall components, etc.) [2]. BOFdat closely follows this logic by dividing the biomass definition process into 3 different steps illustrated in Fig 1. The modular implementation of BOFdat allows users to perform each step independently or in order, following their needs. **Step 1** aims at calculating the stoichiometric coefficients of the major macromolecules mentioned above, thereby providing a computational tool compatible with the experimental methods for the measurement of macromolecular cell composition reviewed by Beck *et al*. [8]. As mentioned below, an important input is the macromolecular weight fraction (MWF) of each category. Also included in the first step is the computation of the growth and non-growth associated maintenance costs from growth data. **Step 2** aims at refining the BOF by adding coenzymes and inorganic ions. The stoichiometric coefficients for suitable metabolites are calculated using the MWF of the soluble pool. This MWF may vary from an organism to another and can be generated experimentally or obtained from the literature. **Step 3** aims at finding condition and species-specific metabolic end goals. To do so, an unbiased approach based on experimental gene essentiality data uses the power of the metaheuristic genetic algorithm (GA) and spatial clustering to identify groups of metabolites that represent cellular objectives under the condition of interest.

Overall, BOFdat is designed to enable modelers to operate each step independently rather than being restrained to a single “do-it-all” function. This design is based on the reality of metabolic modeling where the availability of experimental data is variable and may increase over time. We provide detailed data requirements of each step of the workflow in the “General Usage” section of the documentation (https://bofdat.readthedocs.io/). The characterization of the MWF of the major macromolecules is the most important factor to determine the stoichiometric coefficients and should be prioritized in experimental design. The use of genomic, transcriptomic, proteomic and lipidomic data refines the stoichiometric coefficient under a given condition (S7 Fig), with lipidomic data also informing on the lipid species that should be added to the BOF. To generate accurate growth rate predictions modelers should obtain experimental uptake and secretion rates of major carbon sources and metabolic waste. Lastly, if the modeler’s goal is to maximize the gene essentiality prediction, BOFdat Step 3 can be used along with experimental genome-wide essentiality data to find the metabolites that will optimize this phenotypic prediction.

While strictly following the workflow is not mandatory, this facilitates the mass-balancing of the objective function. Indeed, each addition of a macromolecular category requires the input of its MWF. A fundamental concept for the prediction of growth rate using flux balance analysis is the establishment of a basis such that the product of cell weight by time is equal to 1 gDW/hr [16]. A careful use of BOFdat would ensure that the sum of all MWF is equal to 1, hereby ensuring that the basis for the prediction of growth rate is respected. A tool for the verification of this assumption was previously published and is therefore not included in BOFdat [17].

Finally, while the first step of BOFdat does not require a complete metabolic network, results from the following Step 2 and 3 may be affected by the completeness of the network. We suggest using available tools to fill gaps in metabolic networks [18–22] prior to performing these steps of BOFdat.

### Step 1: Determining macromolecular composition and maintenance costs

**Fig 2:**
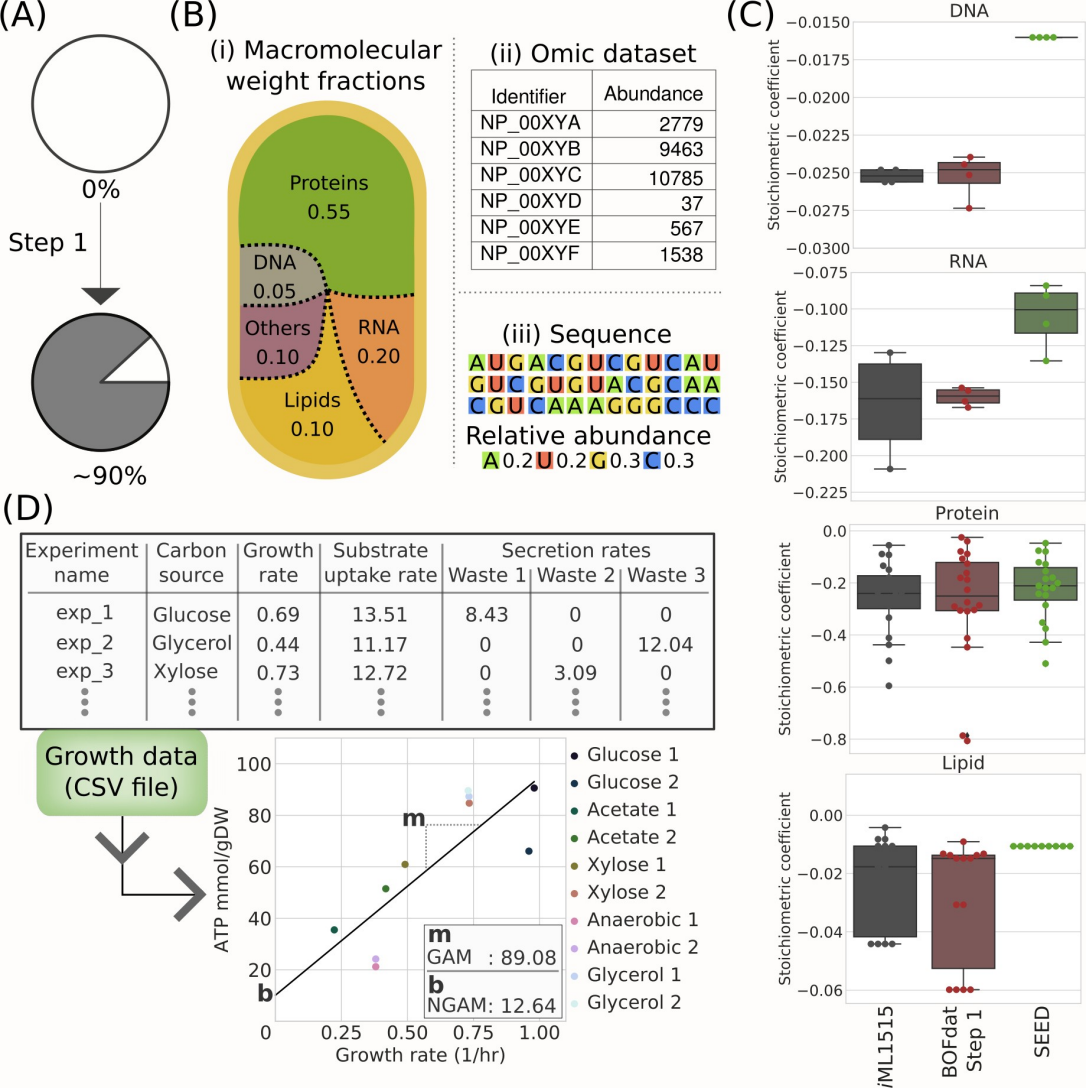
BOFdat Step 1: Calculating the biomass objective function stoichiometric coefficients (BOFsc) for the 4 principal macromolecular categories of the cell. (A) Impact of BOFdat Step 1 on the description of the cellular dry weight. (B) BOFsc are calculated using three data types: i) Macromolecular weight fractions, ii) Omic datasets, and iii) the genome sequence. (C) Comparison of the stoichiometric coefficients used in *i*ML1515 (grey) with those generated by BOFdat (red) and SEED (green). (D) Experimentally measured growth rate, substrate uptake rate, and metabolic waste secretion rate across different conditions are used to constrain the model and generate growth-associated (GAM) and non-growth associated (NGAM) ATP maintenance costs. The GAM is represented by the slope (m) of the linear regression over the conditions, while the NGAM is the Y-intercept (b) of that slope.

In most cells, major macromolecules (DNA, RNA, proteins and lipids) occupy a significant fraction of the total mass [8] (Fig 2A). Quantifying these molecules is thus crucial for a GEM to generate accurate phenotypic predictions. The first step of BOFdat specifically aims at determining the stoichiometric coefficients for each metabolite building block composing these macromolecules. For each molecular category, a BOFdat function requires the input from experimental data (Fig 2). An exhaustive description of the required files and file formats can be found in the docstring of the package and in the documentation (https://bofdat.readthedocs.io/). Implementation and calculation details are provided in S1 Text.

The DNA coefficients are calculated using the genome sequence, which is used to determine the relative abundance of each nucleotide (dATP, dTTP, dCTP, dGTP). The MWF of DNA is provided by the user as a fraction between 0 and 1. The molar weight of each nucleotide is extracted from the model formula weight. Together this information is sufficient to calculate the stoichiometric coefficient (expressed in mmol/gDW/hr) of each metabolite [3] (Fig 2C).

The coefficients of the monomers that compose RNA and proteins are obtained by weighting each metabolite frequency within a sequence (RNA transcript or translated protein) by the relative abundance of this transcript/protein in the corresponding omic dataset. This allows the user to calculate the relative abundance of each ribonucleotide and amino acid (AA) metabolites in the entire cell. To calculate stoichiometric coefficients, BOFdat thus uses the sequence of each RNA and protein along with the relative abundance of each transcript or translated protein. To ensure proper calculation of the stoichiometric coefficients, modelers must provide true relative abundances (i.e. not logarithmically transformed). The whole-cell relative abundances calculated using these input files are then converted into stoichiometric coefficients using the same calculation method as for the DNA metabolites (Fig 2C).

The lipidic constituents of the cell can also be incorporated into the BOF with BOFdat. To do so, modelers require access to a lipidomic dataset. The first input to provide is a conversion between BiGG identifier and the lipid generic name. This conversion step is intended to encourage a lipid network definition that matches experimental data. The second input file contains the relative abundances of each lipid species identified. Once provided with these two elements along with the lipidic MWF, BOFdat applies the conversion to BiGG and finds the molar weight of each lipid to compute their stoichiometric coefficients (Fig 2C). Lipid species may have varying tail length, which may impact their molar weight. In the case where the length of the chain is unknown, BOFdat will use a default R-chain weight that can be changed by the user.

The maintenance costs calculation is also included in the first step of BOFdat. Growth and non-growth associated maintenance (GAM and NGAM) are obtained from growth rate, substrate uptake rate and secretion rate on different media conditions as performed by Monk *et al*. [23] (Fig 2D). While this calculation is included in Step 1, we strongly advise to use a completed (or close to completion) model before generating the maintenance costs. The input file format used to perform the calculation is described in the BOFdat documentation (http://bofdat.readthedocs.io). Briefly, growth-associated maintenance is represented as the slope of the curve obtained by linear regression, and the non-growth associated maintenance is the y-intercept of that curve (S1 Text).

### Step 2: Identifying coenzymes and inorganic ions

**Fig 3:**
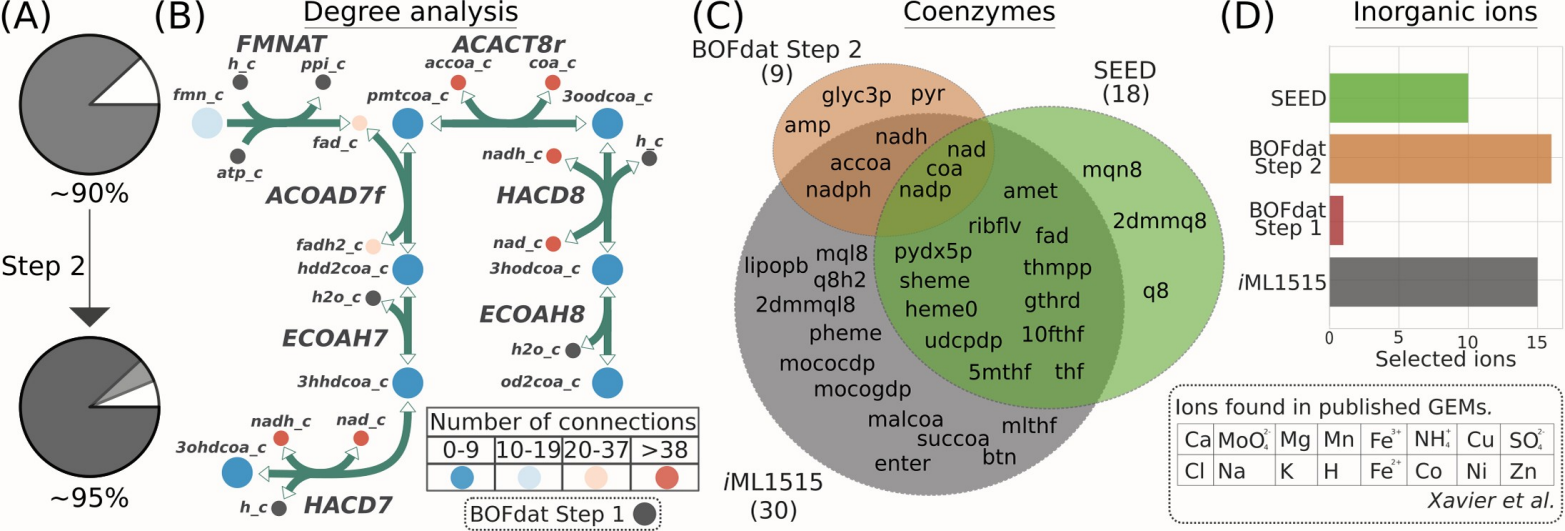
BOFdat Step 2: Identifying and calculating the stoichiometric coefficients of coenzymes and inorganic ions. (A) Pie graphs of the percent dry weight accounted for before and after BOFdat Step 2. (B) The coenzymes found by BOFdat Step 2 are metabolites with a higher degree than the established threshold (S1 Text). Shown is the degree analysis performed on a subset of 7 reactions in *i*ML1515. The metabolites are colored according to the number of reactions to which they participate in the model. Metabolites included in BOFdat Step 1 are removed from the Degree analysis (grey). (C) Venn diagram of the coenzymes found in Step 2 (orange) compared to SEED (green) and the original *i*ML1515 wild-type biomass (grey). Manual curation was used to identify metabolites that qualify as coenzymes in both *i*ML1515 and SEED. (D) Bar chart showing the list of universal ions found by Rocha and colleagues [5] identified in each method. BOFdat Step2 finds the inorganic ions in the model by comparing the model metabolites against this list.

While their mass fraction may not be as significant as the major macromolecules (Fig 3A), coenzymes and inorganic ions composing the soluble metabolite pool are key for cell growth, allowing many enzymatic reactions to take place. Since they are renewed after utilization, coenzymes may participate in many reactions and are often described as ‘currency metabolites’ [24]. The second step of the BOFdat workflow utilizes this property to identify high degree metabolites from the network (Fig 3B), which are considered as *bona fide* coenzymes (S1 Fig).

Xavier *et al*. [5] provided a table of biomass components used in 72 published metabolic models available on the BiGG Models database [25]. This valuable resource was integrated with the BOFdat workflow to add inorganic ions to the BOF since whole-cell experimental identification of inorganic ions is tedious. The 16 identified ions are mapped to the model (Fig 3D). Finding the ions in the metabolic reconstruction signifies their usage by the cell and is hence sufficient to add them to the BOF. To calculate the stoichiometric coefficients for these metabolites, BOFdat uses the MWF of the entire soluble pool. This value may vary from an organism to another, we therefore suggest obtaining the MWF experimentally or extracting the information from organism-specific literature. If this is not possible, BOFdat uses a default weight fraction of 0.05 (5%) of the cell for the entire soluble pool, a value that can be changed by users. The molecules that compose this category are assumed to be represented evenly, and the weight of each molecule is used to obtain the final stoichiometric coefficients, similarly to the method described above (S1 Text).

### Step 3: Identifying organism-specific biomass precursors

**Fig 4:**
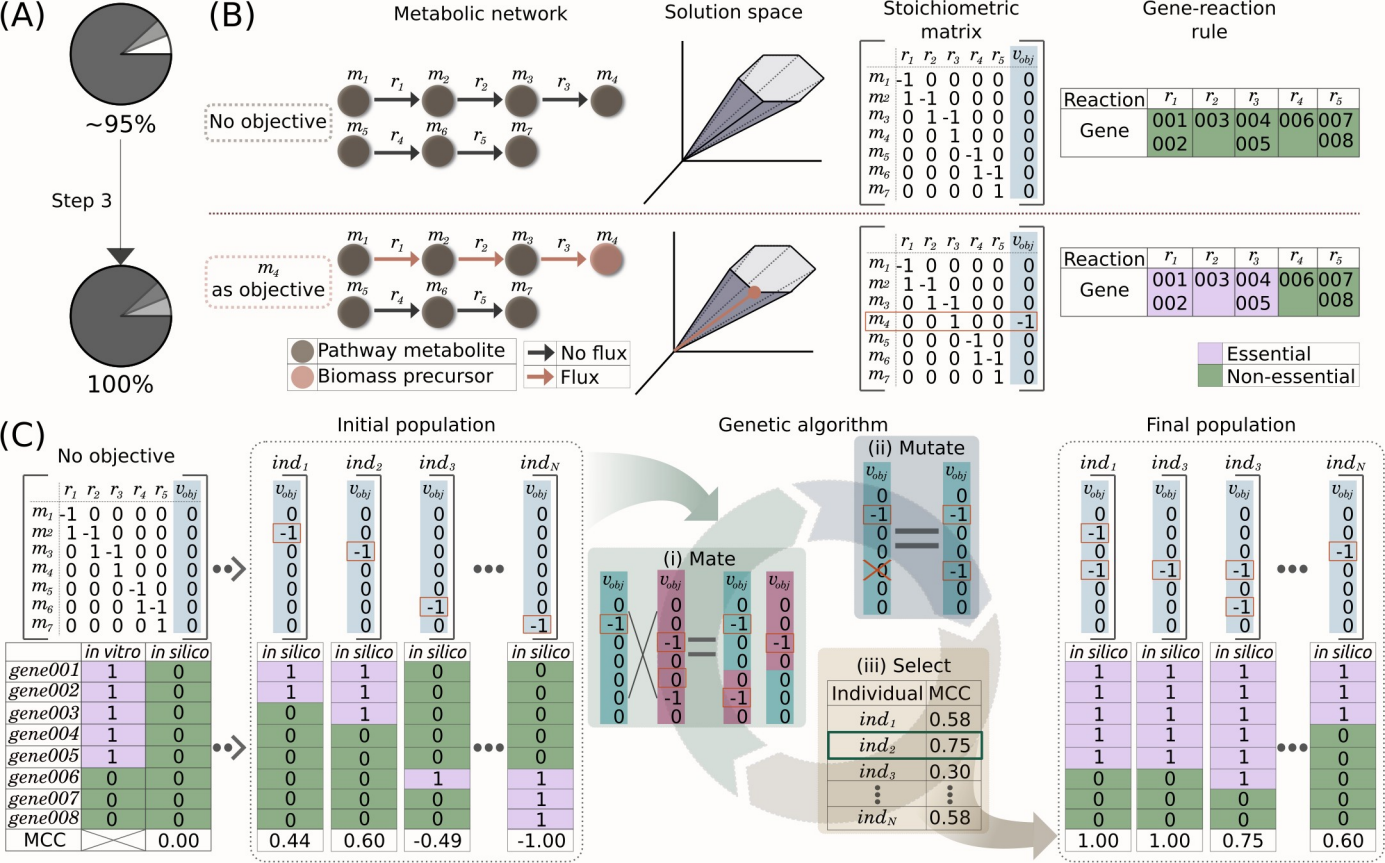
BOFdat Step 3: Identifying species-specific metabolic end goals. (A) After Step 3, the entire weight of the cell is accounted for by BOFdat. (B) Schematic description of the effect of adding a biomass precursor on the prediction of gene essentiality in the model. A simplified metabolic network composed of two linear pathways is depicted with its corresponding stoichiometric matrix S, in which the objective function is presented in the blue column (*v*_*obj*_). The addition of the metabolite *m4* to the objective vector (orange dots and rectangle), forces the flux through reactions *r1*, *r2* and *r3* (orange arrows) and makes genes 001 to 005 computationally essential (purple boxes), defining a new line of optimality in the solution space. (C) Schematic representation of the implementation of the genetic algorithm (GA) using the metabolic network presented in B. The Matthews Correlation Coefficient (MCC) is used to compare *in vitro* (observed) and *in silico* (predicted) gene essentiality data. The MCC is calculated for each individual in the initial population. For simplicity, we represent each individual with a single biomass component. The genetic operators (mate, mutate and select) are then applied on a population to generate new individuals with higher MCC values (used here as a measure of fitness). At the end of the evolution, the final population is composed of different individuals with mainly high MCC values.

The addition of the major macromolecules combined with the coenzymes and inorganic ions allow the first two steps of BOFdat to represent a high fraction of the cell weight (~95% for *E*. *coli*, Fig 4A). Using experimental gene essentiality data, BOFdat Step 3 aims at identifying condition and species-specific biomass precursors. These remaining metabolites are likely to vary from one species experimental condition to another, hence their addition via an unbiased approach ensures a context-specific composition of the BOF. BOFdat identifies these biomass precursors through multiple iterations of a genetic algorithm (GA). The implementation of the GA is based on the DEAP toolbox version 1.2 [26] as described in S1 Text.

#### Concepts underlying the implementation of the GA

The GA supposes that the addition of a metabolite to the objective function may change and improve the gene essentiality prediction of a model. As illustrated in a simplified network composed of two linear pathways (Fig 4B), the addition of a metabolite to the objective function sets a line of optimality on the solution space. To satisfy the new objective set by the addition of metabolite *m4* to the biomass, flux must go through reactions *r1*, *r2* and *r3*. Since *m4* can only be produced through these reactions, the model predicts them as essential along with the genes to which they are associated by the gene-protein-reaction rule (GPR). The COBRApy toolbox [27] allows users to generate model-wide single gene deletion predictions where each gene in the model is removed individually and the resulting growth is assessed by attempting to solve the model. A growth/no-growth phenotypic prediction can then be generated for every gene in the model similar to high-density transposon mutagenesis experiments [28] and other high-throughput approaches to assess gene essentiality *in vivo* [29]. For comparison purposes, the gene essentiality observations and predictions can be converted into binary vectors, enabling the use of common distance metrics for their comparison. The Matthews Correlation Coefficient (MCC) is a metric frequently used to evaluate GEMs’ gene essentiality prediction, as it takes account of false and true positive and negative observations in a balanced way, and works with binary classifications [30]. Using this metric, the gene essentiality prediction resulting from a newly formulated BOF can be evaluated against experimental data, where an MCC equal to 0 would be equivalent to random whereas a MCC of 1 is an exact match between predictions and observations (Fig 4C). This concept allows users to define the main elements of a genetic algorithm where each newly generated BOF is defined as an individual and the MCC score can be used as a fitness metric. To ease the usability, BOFdat divides Step 3 into three different operations: 1) a group of individuals called an “initial population” is generated; 2) the GA is applied to the initial population by iteratively applying genetic operators to its individuals in a process termed *in silico* evolution, referred to simply as evolution throughout the text; and 3) the results are interpreted through spatial clustering to form metabolic end goals.

#### Definition of the initial population

An initial population is generated, on which the evolution will be performed. Conceptually, each individual in the population may contain any combination of all metabolites in the model. In order to reduce the search space of the algorithm, BOFdat utilizes a series of feature selection operations. The metabolites from the output of BOFdat Step 2 are removed from the complete set of metabolites. Metabolites that cannot be produced individually by the model are also removed. Lastly, the impact on gene essentiality prediction for remaining metabolites is assessed by optimizing for the production of each individual metabolite and calculating the MCC score as described above (S2 Fig). Metabolites with resulting MCC scores above a defined threshold are selected as input to Step 3 and referred to as the metabolites subset (S1 Text).

An individual is represented by a binary list referring to the presence (1) or absence (0) of the metabolites subset (Fig 4C). Individuals in the initial population are randomly generated to maximize diversity and coverage of the selected metabolites. It has been previously shown that the outcome of an evolution may be affected by the initial population [31]. Therefore, it is recommended to perform multiple evolutions. BOFdat hence requires the user to input the desired number of initial populations so that one evolution per generated initial population can be performed (S3 Fig). The suggested number of evolutions to perform is discussed in the results section.

#### Application of the GA

An evolution is performed by iteratively applying genetic operators to the initial population. The three genetic operators used in the GA are mutation, mating, and selection. Mating and mutation operators generate diversity within the population, allowing the GA to screen more combinations of BOFs. During an *in silico* mating event, crossovers can arise between two individuals. In such case, a position in the list of two individuals is chosen and their elements are exchanged. An individual is an index of metabolites associated with an indicator of presence (1) or absence (0). This format allows to apply mutations which revert a 0 into a 1 and vice-versa. Selection is key to the GA since it ensures that a trait, referred to as fitness, is optimized throughout the evolution. In BOFdat Step 3, the fitness of each individual is measured by the MCC score, where a higher MCC score increases the chances of an individual being a member of the next generation. The process of applying the genetic operators on a population of individuals is called a generation.

The GA implementation also ensures that a limited number of metabolites is contained within individuals. To do so, the number of metabolites that a single individual can contain is restrained by applying a second objective to the fitness function used to select individuals. This maximizes the MCC score while minimizing the number of selected metabolites (S4 Fig and S1 Text). As mentioned in the DEAP documentation (https://deap.readthedocs.io), the output of the GA can be presented in the form of a logbook or hall of fame (HOF). The logbook records the statistics of the population fitness at each generation (mean, maximum and minimum on the individuals’ fitness), allowing the user to follow the increase of fitness over generations. The HOF records the best individuals generated throughout an entire evolution and can be used to assess the metabolite content of optimal solutions.

#### Interpreting the result of multiple evolutions

**Fig 5:**
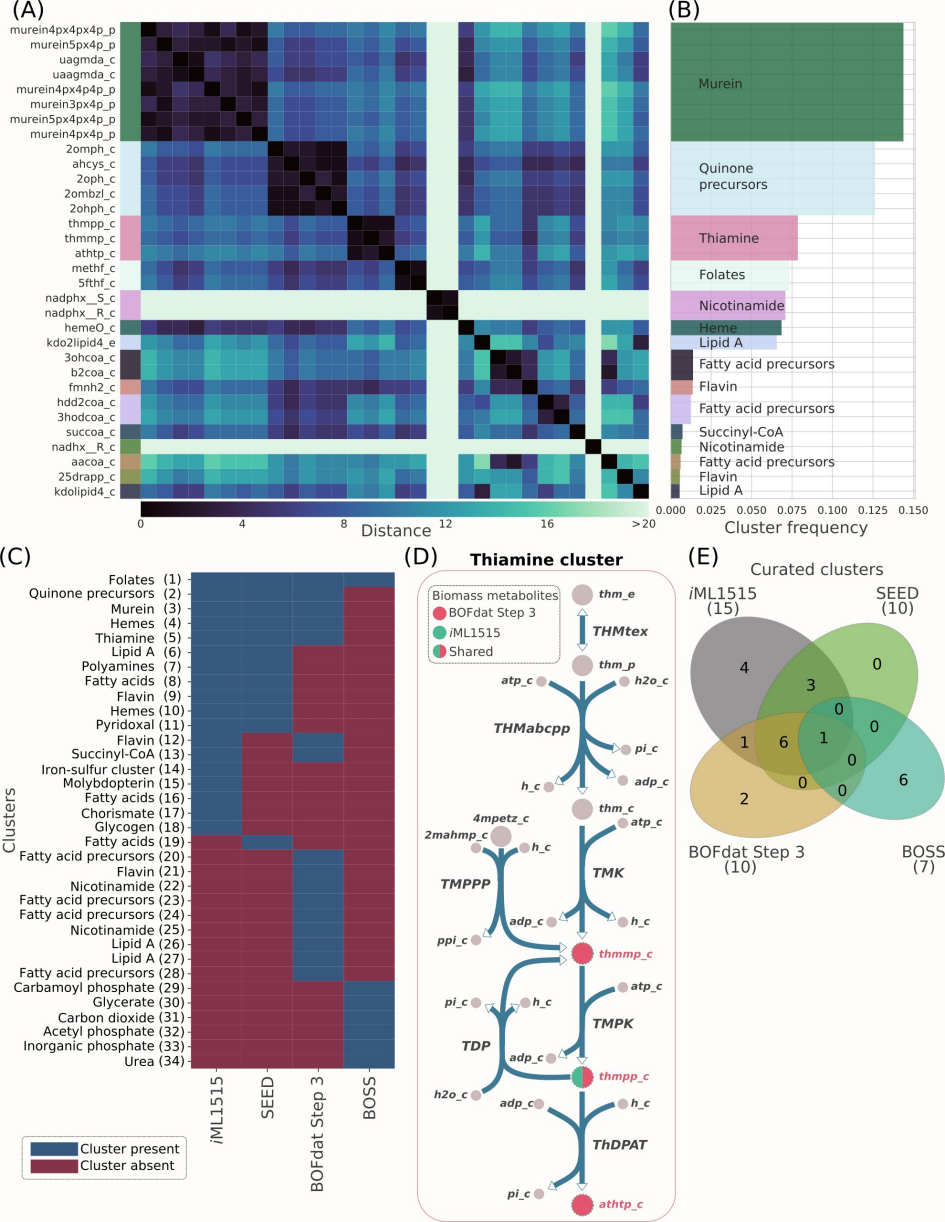
Identification of metabolic end goals by BOFdat Step 3. (A) The distance matrix for the metabolites selected based on their individual occurrence in HOFs for 150 different evolutions were clustered using DBSCAN (eps = 8, S1 Text) (S6 Fig). (B) Each of the 15 clusters identified in A were named by manually identifying the most frequent metabolite ontology in EcoCyc. The cluster frequency is the sum of each individual metabolite frequency within the cluster. (C) The metabolites from the original *i*ML1515, SEED, BOFdat and BOSS are pooled together, and clustered based on network distance using DBSCAN (eps = 10, S1 Text). The clusters that are present (blue) and absent (red) for a given method are identified. The naming of the clusters was performed manually and the metabolite composition of each of them is available in S2 File. (D) Metabolic map of the thiamine cluster identified in B. The metabolites selected by BOFdat (red) are within 2 reactions of each other, and the biomass component from *i*ML1515 (green) lies in the middle. (E) Clusters shown in C were curated to group together those representing the same end goals, and presented as a Venn diagram.

The results of multiple evolutions are interpreted through the clustering of significant metabolites identified by the GA based on their network distance (S1 Text). The stochasticity involved in the application of the genetic operators combined with the generation of multiple evolutions provides many optimal solutions with similar or equal fitness that nevertheless differ in metabolite content (S5 Fig). Obtaining the same MCC score for different individuals is conceptually possible since the addition of different metabolites to the BOF may trigger the same gene essentiality prediction. Conversion of optimal results into biological knowledge is therefore critical for modeling work since modelers ultimately need to decide which metabolites to add to their BOF. BOFdat provides a way to interpret the GA results by applying spatial clustering to the HOF of combined evolutions. The distance matrix is obtained by calculating the shortest path between metabolites in the network (S1 Text). Clustering of the distance matrix is performed with the DBSCAN algorithm for a list of significant metabolites selected based on their frequency of appearance in the HOF of the combined evolutions (Fig 5A, S6 Fig). The term “species-specific metabolic end goal” is applied to describe these clusters as they link biological knowledge provided by the network reconstruction to relevant end goals found with the GA (Fig 5B).

## Results and discussion

**Fig 6:**
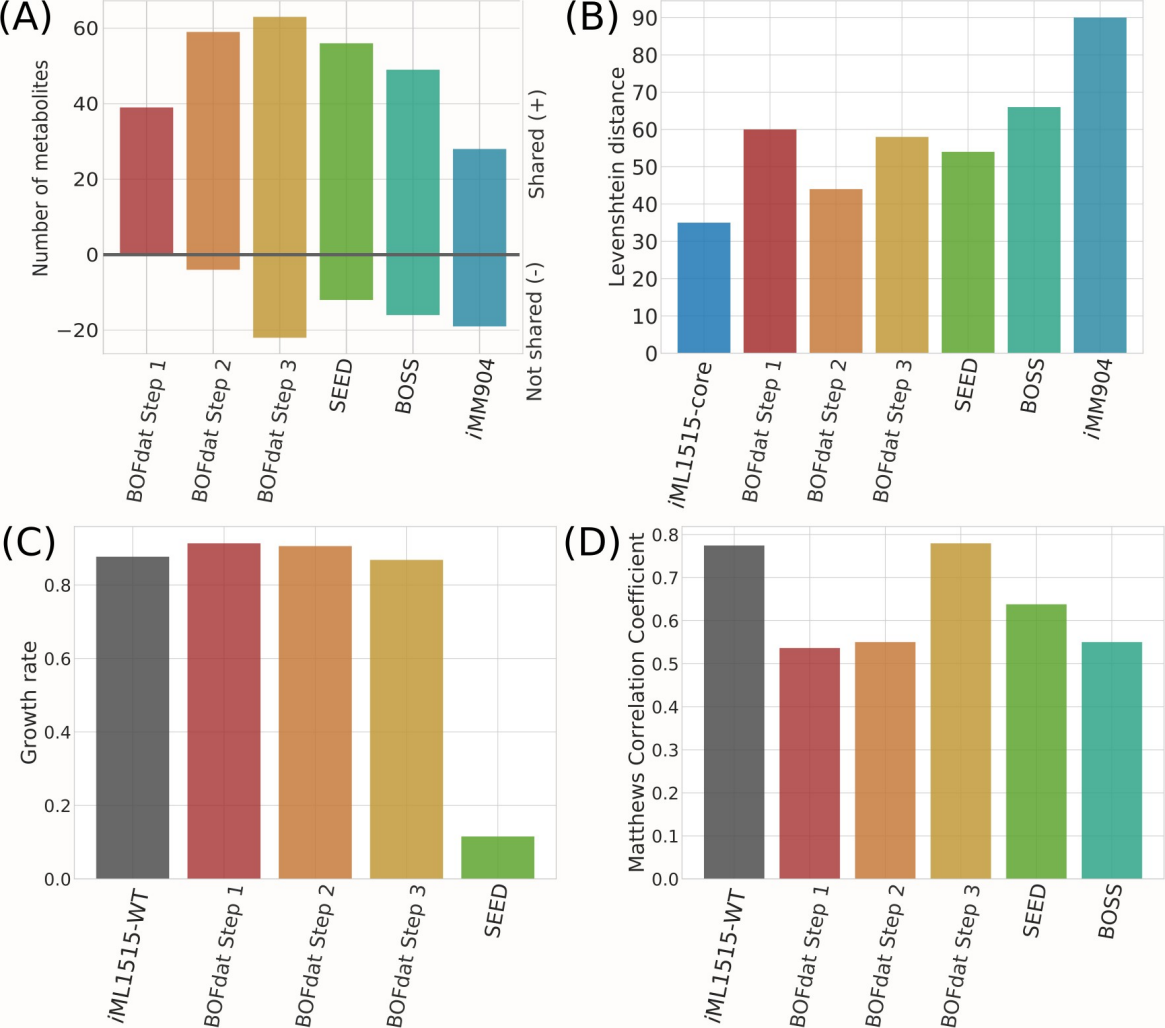
Comparison of phenotypic predictions and metabolite composition between the three steps of BOFdat, the original *i*ML1515 BOF, SEED, and BOSS. (A) Number of metabolites shared with the *i*ML1515 wild-type (WT) biomass (positive values), and specific to each of the method (negative values). (B) Levenshtein distance calculated between the original *i*ML1515-WT biomass and the BOF generated by each of the other methods, as well as with the *i*ML1515-core and the yeast model *i*MM904 BOF. The Levenshtein distance represents the number of additions, subtractions or substitutions that need to be applied on the list of metabolites from the compared BOF to retrace the reference BOF. (C) The predicted growth rates for all Steps of BOFdat are compared to the *i*ML1515-WT and SEED. BOSS imposes a fixed growth rate as part of the optimization problem and was hence not compared since it does not formulate a prediction. (D) Gene essentiality prediction as evaluated with a Matthews correlation coefficient when compared with experimental data generated on glucose minimal media [2].

To validate BOFdat, we reconstructed the BOF of *Escherichia coli* K-12 MG1655. We compared our obtained biomass compositions and phenotypic predictions with the recently updated *E*.*coli* model *i*ML1515 [23] and with two available methods to generate BOF: SEED and BOSS. The phenotypic predictions were formulated by setting the method’s biomass composition as the new objective and FBA was performed. SEED is an automated platform for genome-scale metabolic reconstruction from genome information [9]. When generating a model with SEED, the user decides the biomass composition of the organism of interest from a set of predefined BOFs, based on phylogenetic relationship. For our purpose, we chose the gram-negative bacterial BOF and converted the obtained SEED metabolite identifiers to BiGG identifiers with MetaNetX [32] (S1 File). The metabolites for which no correspondence was obtained were manually converted. The Biological Objective Solution Search (BOSS) [11] algorithm uses 13C-based flux analysis data (MFA) to infer the stoichiometric coefficients for a new column of the stoichiometric matrix, which defines the BOF. In contrast to the SEED that uses *a priori* knowledge to determine the BOF composition and thus can be categorized as a biased method, BOSS uses a mixed-integer linear programming (MILP) optimization to extract biomass precursors and components from experimental data and is considered unbiased. In order to provide a valid point of comparison with the unbiased third step of BOFdat, we used an expanded version of the original BOSS that performs the optimization at the genome-scale (S1 Text) [33]. GEMs have been shown to efficiently predict cellular phenotypes such as growth rate and gene essentiality [34]. As illustrated in Fig 6A and 6B, BOFdat provides a method to ensure that the BOF resulting from its use provides predictions as close as possible to experimental measurements.

### Using omic datasets with macromolecular weight fractions allows to accurately calculate stoichiometric coefficients

The stoichiometric coefficients for four categories of major macromolecules were calculated using BOFdat (Fig 2C). We validated the use of omic datasets to calculate stoichiometric coefficients by comparing BOFdat generated stoichiometric coefficients against the original *i*ML1515 biomass and SEED. Fig 2C shows the stoichiometric coefficients for the four categories of macromolecules obtained from *i*ML1515, SEED or calculated with BOFdat Step 1. For all macromolecular categories, the median of the stoichiometric coefficients calculated with BOFdat is closer to the original *i*ML1515 median than the one obtained using SEED.

Given the availability of experimental data, BOFdat can be used to calculate stoichiometric coefficients for varying conditions. Here we used a high-quality quantitative proteomic dataset [35] to calculate the stoichiometric coefficients of each amino acid under 18 different conditions (S7 Fig). For each condition, this dataset includes the MWF of proteins and quantitative proteomic measures, allowing to compare the impact of both parameters on the determination of the stoichiometric coefficients. We found that the MWF is significantly more important than the omic dataset itself (Pearson *r =* 0.984, *p-value* = 3.610e-14) (S1 Text). This result should incite modelers to query precise determination of the cell’s composition under the studied condition [8]. This result also implies that, in a case where expression data is unavailable, BOFdat can still be used to calculate stoichiometric coefficients by replacing the relative abundance of each transcript/expressed protein by equal values (e.g. 1).

### BOFdat identifies biomass precursors as clusters of metabolites

BOFdat Step 3 generates BOFs that allows the model gene essentiality prediction to match experimental data optimally. In our case study, the MCC of the generated BOF equaled or exceed that of the original *i*ML1515 wild-type BOF (Fig 6B, S3 Fig). However, the metabolite content varies from an optimal solution to another, a consequence of the stochasticity involved in the GA [31]. In order to generate a comprehensive and robust solution usable by modelers, BOFdat performs a final step of interpretation that involves clustering relevant metabolites, i.e. those that were frequently found in individuals with higher fitness, based on their relative distance in the metabolic network. The rationale supporting the clustering approach is that neighboring metabolites in the network may represent a single metabolic objective.

To evaluate the approach we generated 150 different initial populations and evolved each of them for 500 generations. The optimal solutions from those 150 evolutions were filtered and clustered as described above (S1 Text and S6 Fig) and 15 clusters were identified. The distance matrix (Fig 5A) allows discerning distinct clusters supporting the rationale that neighbor metabolites in the network represent metabolic objectives. The metabolite frequency is the number of times a metabolite appeared in an individual over the number of individuals with higher fitness. The cluster frequency is defined as the sum of all metabolite frequencies within this cluster. In Fig 5B, the clusters were ranked by order of cluster frequency and manually named according to the most representative ontology found on EcoCyc [36] (https://ecocyc.org/). Naming the clusters creates a link from identified metabolites to knowledge, where the name of a cluster represents a metabolic objective.

We describe those objectives and their importance for the first 3 clusters identified by order of cluster frequency. The first cluster represents the murein content of the cell, a key cell wall component of *E*. *coli* that maintains bacterial shape and allows resistance to intracellular osmotic pressure [37]. The second cluster is composed of metabolites involved in ubiquinone biosynthesis, also known as coenzyme Q10 (1,4-benzoquinone), critical for cellular respiration [38]. While both SEED and *i*ML1515 identified the production of quinone as biomass objective (Fig 5C), the metabolites chosen to represent it are different (see metabolites specific to SEED, Fig 3C). The fact that different metabolites may represent the same biomass objective and create pseudo-diversity amongst BOFs is a concern raised by Rocha and colleagues [5]. We suggest that clustering metabolites based on network distance provides a solution to this recurrent issue. The third cluster is composed of thiamine (vitamin B1) related compounds, an essential coenzyme in *E*. *coli* [39] (Fig 5D). This cluster demonstrates how, in practice, BOFdat identifies metabolites in a linear pathway as demonstrated in Fig 4B. BOFdat identified 3 different metabolites in this pathway and one of them is present in *i*ML1515 BOF. Together, these observations support the fact that the identified clusters are consistent with current biological knowledge, with the more frequent clusters being species-specific essential components. Moreover, BOFdat provides an approach for the selection of biomass precursors and components that is condition-dependent and actively represents cellular objectives. Perhaps this type of information can be useful to modelers because it yields data-driven information on the cell’s composition and its metabolic objectives, a challenge that was formulated before [12,13].

### Benchmarking the clustering approach

We compared the metabolites found with BOFdat by applying spatial clustering to the complete list of metabolites found in each method (Fig 5C). In total 34 clusters were identified of which 18 associated with *i*ML1515, 12 with SEED, 16 with BOFdat and 7 with BOSS. These raw results revealed that SEED is the closest method to *i*ML1515 with 11 shared clusters, while BOFdat shares 7 and BOSS shares 1. The naming of the clusters found for each method was executed in the same way as for BOFdat alone (Fig 5B) and clusters sharing the same name were grouped together since they represent the same metabolic objective (Fig 5E). While SEED and *i*ML1515 share more biomass objectives together than the unbiased computational methods, BOFdat shares more curated clusters with *i*ML1515 (8) than BOSS (1). The number of non-shared clusters is also lower for BOFdat (2) than for BOSS (6). The underperformance of BOSS to identify biomass precursors is likely to be attributed to the coverage of the data used to generate the predictions. While state-of-the-art MFA data was used for the prediction [40] (S1 Text), the number of covered reactions only reached ~60. As noted previously [11], MFA typically uses simplified models of central metabolism with lumped reactions, as is the case with the MFA data used here. Genome-wide gene essentiality data used by BOFdat hence circumvents these known issues of inferring metabolic objectives from MFA [11].

The two clusters specific to BOFdat are nicotinamide and fatty acid precursors. Nicotinamide was found in Step 2 (Fig 3C), but the damaged hydrated forms (nadhx and nadphx) were identified by BOFdat Step 3. Fatty acid precursors were identified despite the fact that lipids were identified in Step 1. These specific metabolic objectives are consistent with other methods (Fig 5) and previous Steps of BOFdat.

BOFdat Step 3 aims to facilitate the identification of metabolic objectives by modelers by providing a data-driven way of selecting metabolites. An automated final solution is suggested by BOFdat following the clustering into biomass objectives (S1 Text). To provide users with recommendations on usage, we analyzed the impact of the number of evolutions on 1) the resulting MCC scores of these automated final solutions and 2) the number of clusters that were shared with the original *i*ML1515 biomass (S8 Fig). The impact of hyper-parameters on the clustering algorithm were also studied (S9 Fig, S1 Text). In brief, while the number of shared clusters steadily increased from 10 to 150 evolutions pooled together, the optimal MCC score could be reached after 20 evolutions (S8A Fig). A single evolution of 500 generations required ~480 hours (~21 hours on a 24 cores compute node) with a memory peak of ~10GB of RAM. To fulfill the requirements mentioned above (20 evolutions for 166 generations) would require ~3360 hours.

### BOFdat generates biomass objective functions that recapitulate key model predictions

We characterized the impact of each step of the procedure of BOFdat by evaluating the phenotypic prediction after each step and comparing with SEED and BOSS (Fig 6). We compared the metabolite content of all methods to the *i*ML1515-WT BOF (Fig 6A). As a negative control, we used the BOF from a distant organism, using the yeast model *i*MM904 [41]. The number of metabolites that were shared with the *i*ML1515 BOF increased at each step. While the number of shared metabolites is higher for BOFdat Step 3 than any other step or method, the metabolites that were not shared also increased, as expected, since BOFdat finds biomass objectives in clusters of metabolites (Fig 5). To account for correct additions, missed metabolites or wrongly added ones, we calculated the Levenshtein distance (S1 Text) of each biomass composition to the *i*ML1515-WT BOF (Fig 6B). This distance allows one to compare lists of different sizes which is the case for all BOF presented here. A smaller Levenshtein distance means less modifications need to be applied to trace back to the reference. The Levenshtein distance is minimized in Step 2 (from 60 to 44), by the addition of inorganic ions and coenzymes. The distance increases when adding the metabolites from Step 3 chosen after spatial clustering and the automated selection (58), slightly higher than SEED (54) but lower than the other unbiased approach BOSS (66). The predicted growth rate (Fig 6C) generated by BOFdat (0.868 h-1) is closer to the original prediction (0.877 h-1) than the one from SEED (0.116 h-1). This prediction is likely to be attributed to a correct prediction of GAM and NGAM costs. The BOSS approach was not compared for growth rate as the coefficients obtained with it requires fixing the growth rate to a certain score. The gene essentiality prediction was assessed using Matthews Correlation Coefficient (MCC, Fig 6D) and step three of BOFdat (MCC = 0.779) was found to be higher than the original prediction (MCC = 0.775) as well as both SEED (MCC = 0.638) and BOSS (MCC = 0.550). The performance advantage of BOFdat on that aspect is explained by the fact that the genetic algorithm (Step 3) specifically minimizes the distance from experimental data (Fig 4). Indeed, Steps 1 and 2 obtained MCC scores of 0.536 and 0.550, respectively. Together, these elements suggest that the definition of a BOF from experimental data with BOFdat provides acceptable phenotypic predictions that are in par or exceed existing methods.

## Availability and future directions

BOFdat is a systematic computational framework for the definition of biomass objective functions for genome-scale models from experimental data. BOFdat is implemented in Python and is conceived to be a part of the COBRApy ecosystem [27], an environment commonly used to build and analyze GEMs. Our package is accessible for installation through the Python package index (PyPI). The open-source code as well as an example usage and required input files are available on GitHub (https://github.com/jclachance/BOFdat) where a link to the full documentation and API is available. We are confident that its use will contribute to more reliable BOF and GEMs. By providing an unbiased, data-driven approach to defining biomass objective functions, BOFdat has the potential to improve the quality of new genome-scale models and also greatly decrease the time required to generate a new reconstruction. With the increasing number of omic datasets being generated and the realization of community models, we expect BOFdat to be leveraged for the generation of condition- and strain-specific BOF definitions, hereby increasing the quality of GEMs phenotypic predictions. While using experimental essentiality data provides metabolic end goals, other unbiased methods using different data types could be developed and added to the package, hence revealing more precisely the metabolic end goals of the cells.

## Supporting information

S1 Fig Distribution of the metabolites degree in the *E*. *coli* metabolic network used to identify coenzymes (BOFdat Step 2). As established in the literature, the degree is the number of metabolites to which a given metabolite is linked in the network. The red dashed line represents the threshold to identify a coenzyme, set as the mean (5.63) + one standard deviation (33.26) of 1877 metabolites of the network.
(TIF)

S2 Fig Distribution of individual MCC values for metabolites. The pre-feature selection for the generation of initial populations of BOFdat Step 3 includes the evaluation of the MCC value of each remaining metabolites (dark blue bars). The *i*ML1515 model contains 1877 metabolites, 63 metabolites were removed because they were present in BOFdat Step 2 and 766 were removed because the model could not solve when they were set as an objective. The MCC was determined for the 1048 remaining metabolites. The threshold is set at the mean of the distribution + one standard deviation (MCC = 0.20) allowing to select 186 metabolites with the highest potential and referred to as the metabolites subset.
(TIF)

S3 Fig Distribution of the genetic algorithm output for 150 evolutions over 500 generations. At each generation, the highest MCC value of the population was plotted for each evolution (left Y-axis). The solid horizontal red line represents the baseline established by the MCC value of the original *i*ML1515 BOF (0.775). The orange dashed line represents the percentage of evolutions in which the best individual exceeds the baseline (right Y-axis). The vertical green line shows the number of generations necessary to obtain 50% of evolutions exceeding the baseline, in this case 166.
(TIF)

S4 Fig Impact of the size constraint in the genetic algorithm. Number of metabolites contained in each individual (Y-axis) present in every generation over a typical evolution. The size constraint is a second weight applied on the fitness function. Here a weight of 1.0 is applied to maximize the MCC while a weight of 0.0 (A) and -0.25 (B) is applied to evolutions performed for 200 generations. In both cases, the individuals are initialized with a number of metabolites randomly varying from 60 to 100. Without applying a size constraint (A) the size of the individuals is ever increasing through the evolution. The application of a weight of -0.25 penalizes the individuals for the addition of metabolites and the individual size is stabilized (B).
(TIF)

S5 Fig Multiple correspondence analysis (MCA) of individuals generated with BOFdat Step 3. MCA is used to compare feature similarity between 35 individuals selected from HOFs from different evolutions. The fitness value is represented from the minimum value recorded (0.7453, blue) to the maximum (0.7716, red). The percent variability explained by the first two factors is 19.54% for factor 1 and 13.92% for factor 2. This analysis shows that similar fitness values can be obtained from diverse sets of metabolites.
(TIF)

S6 Fig Schematic description of spatial clustering in BOFdat Step 3. Each evolution generates a Hall of Fame (HOF) that, by default, stores the 1000 best individuals generated in the course of that evolution. The individuals produced by all the evolutions are pooled together and, arbitrarily, the 20% best individuals are selected based on their fitness value. The metabolites that compose these individuals are then ranked based on their frequency of apparition in those individuals. The metabolites with a frequency of apparition above average are selected. The Dijkstra algorithm is then used to calculate the distance in the metabolic network between the selected metabolites, assuming that a single reaction is one unit of distance. The distance matrix is then clustered using DBSCAN to identify clusters of metabolic end goals. The removal of highly connected metabolites may break the connection of certain metabolites to the rest of the network. For visualization purposes, the maximum distance is attributed to such metabolites.
(TIF)

S7 Fig BOFdat Step 1 allows calculating stoichiometric coefficients under different experimental conditions. A high-quality quantitative proteomic dataset was used to generate stoichiometric coefficients for 18 different growth conditions [35]. Each boxplot represents the BOFsc percent difference of each amino acid (AA) in a given condition compare to glucose (left). The dataset also includes the macromolecular weight fraction (MWF) of protein in the cell for each condition. The histograms represent the difference between the protein weight fraction of each condition from the same dataset, against the fraction determined in the glucose growth condition (right). This shows that the stoichiometric coefficients are mainly affected by the MWF.
(TIF)

S8 Fig Impact of the number of evolutions on the clustering results. HOFs from the 150 evolutions over 500 generations are sampled, such that 20 samples are formed per number of evolutions pooled together (x-axis). The number of evolutions is the number of HOF pooled together to form clusters and select metabolites. (A) The MCC value of the automatic selection of metabolites following the clustering in BOFdat Step 3 for an increasing number of HOF from different evolutions. The median MCC value exceeds the baseline set by *i*ML1515 for 20 evolutions pooled together and reaches a maximum of 0.779 after 40 evolutions. (B) The selected metabolites are compared against the original biomass of *i*ML1515. New clusters are formed and the distribution of percentage of shared clusters is shown (y-axis). The use of more evolution yields an increase in the percentage of shared clusters with the original BOF from 26.61% for 10 evolutions to 31.19% at 150 evolutions.
(TIF)

S9 Fig Impact of hyperparameters on the clustering results. Selected metabolites from the 20% best individuals generated over 150 generations were clustered using the DBSCAN algorithm while varying the hyperparameters (eps and min_sample). As shown in the legend, two different values of min_sample were used (0.5 in blue, and 1.5 in orange). For these two min_sample values, the eps was varied from 1 to 50 and the number of clusters generated for these hyperparameters recorded (y-axis). Varying the “min_sample” for values 0 to 1 generated a single curve, while using a min_sample above 1 generated another. Hence only two values were represented. In this study, we systematically used a min_sample value inferior or equal to 1 (blue curve) and eps values that yielded a number of clusters around half of the number of metabolites provided.
(TIF)

S1 File SEED metabolite identifiers of the gram-negative bacterial BOF converted to BiGG identifiers. (CSV)

S2 File Description of clusters for Fig 5C. (CSV)

S1 Text Supplementary methods and implementation details. (PDF)
