# Supplementary figures and images for: BOFdat: Generating biomass objective functions for genome-scale metabolic models from experimental data
Source: PLoS Comput Biol. 2019 Apr 22;15(4):e1006971. doi: 10.1371/journal.pcbi.1006971 (PMC6497307; doi:10.1371/journal.pcbi.1006971)

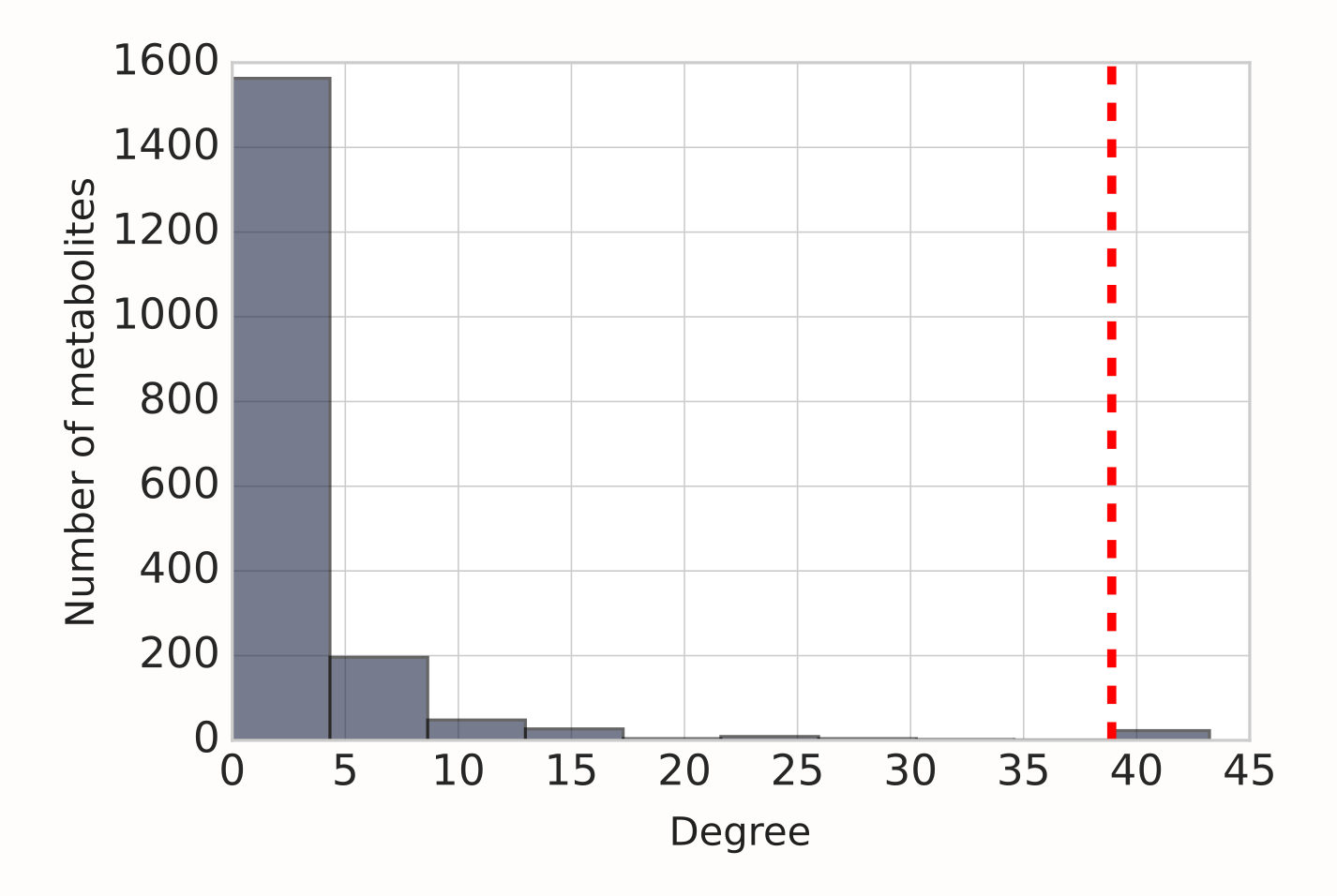

Supplement: S1 Fig — As established in the literature, the degree is the number of metabolites to which a given metabolite is linked in the network. The red dashed line represents the threshold to identify a coenzyme, set as the mean (5.63) + one standard deviation (33.26) of 1877 metabolites of the network. (TIF) [file pcbi.1006971.s001.tif]

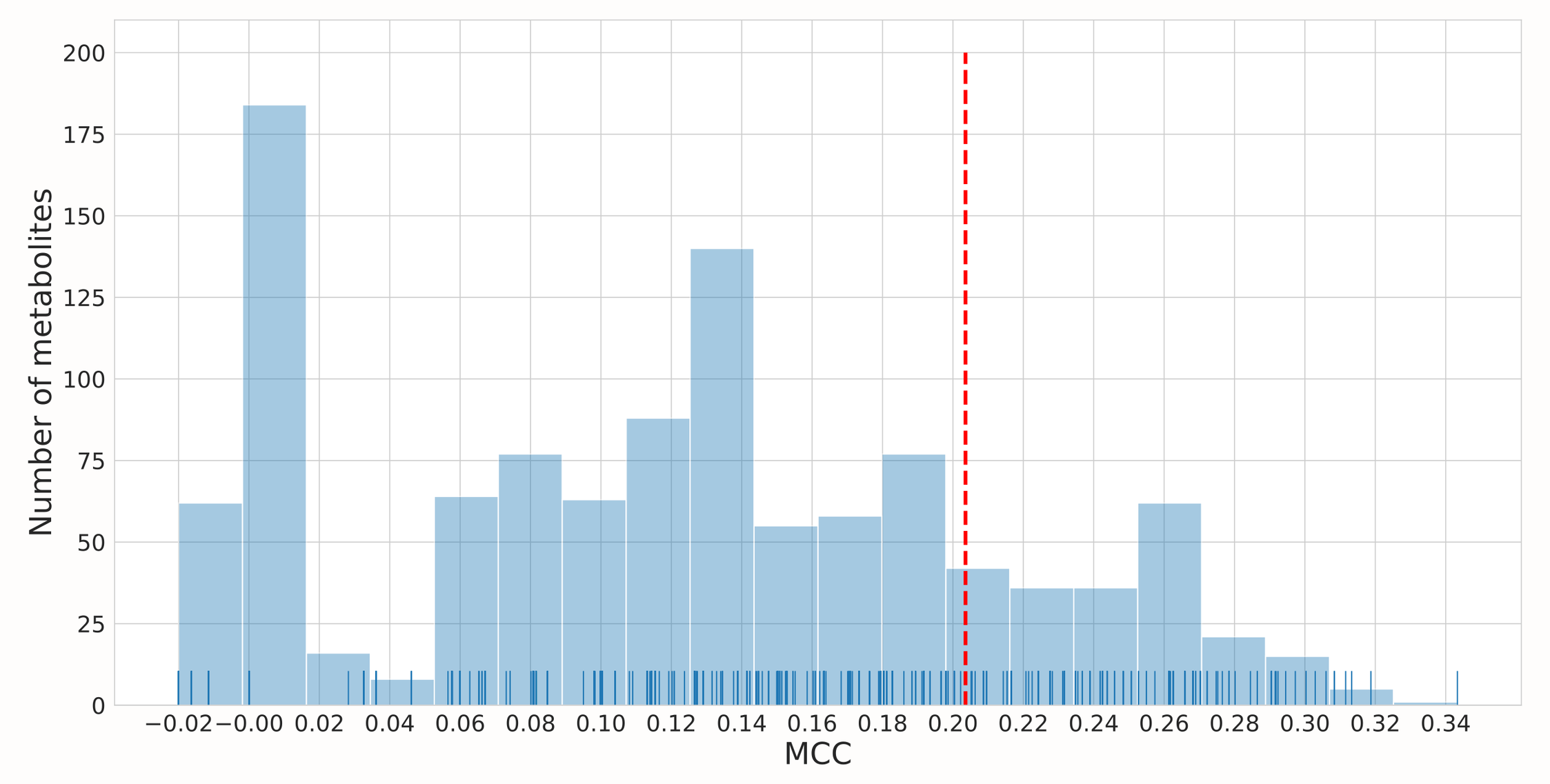

Supplement: S2 Fig — The pre-feature selection for the generation of initial populations of BOFdat Step 3 includes the evaluation of the MCC value of each remaining metabolites (dark blue bars). The iML1515 model contains 1877 metabolites, 63 metabolites were removed because they were present in BOFdat Step 2 and 766 were removed because the model could not solve when they were set as an objective. The MCC was determined for the 1048 remaining metabolites. The threshold is set at the mean of the distribution + one standard deviation (MCC = 0.20) allowing to select 186 metabolites with the highest potential and referred to as the metabolites subset. (TIF) [file pcbi.1006971.s002.tif]

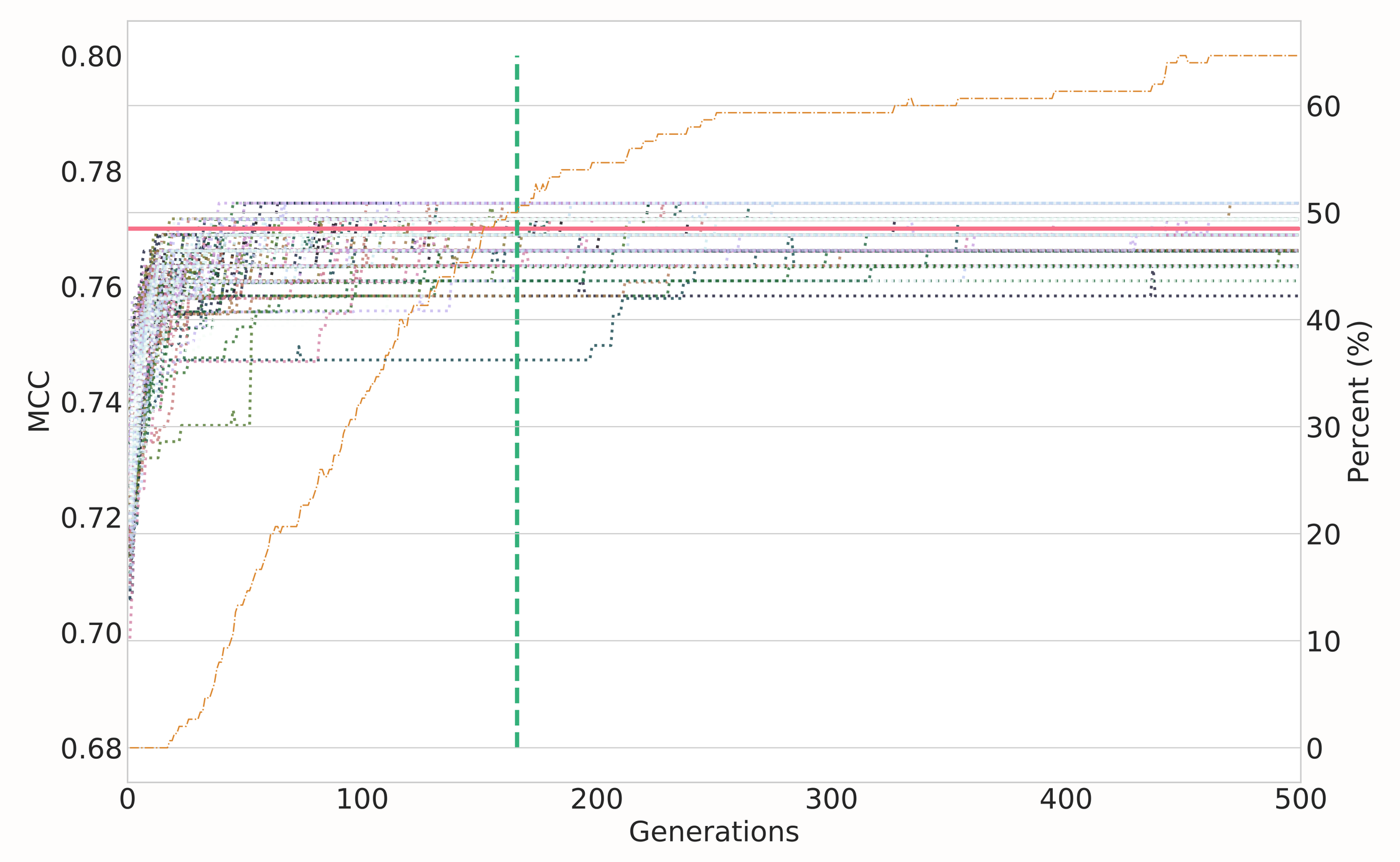

Supplement: S3 Fig — At each generation, the highest MCC value of the population was plotted for each evolution (left Y-axis). The solid horizontal red line represents the baseline established by the MCC value of the original iML1515 BOF (0.775). The orange dashed line represents the percentage of evolutions in which the best individual exceeds the baseline (right Y-axis). The vertical green line shows the number of generations necessary to obtain 50% of evolutions exceeding the baseline, in this case 166. (TIF) [file pcbi.1006971.s003.tif]

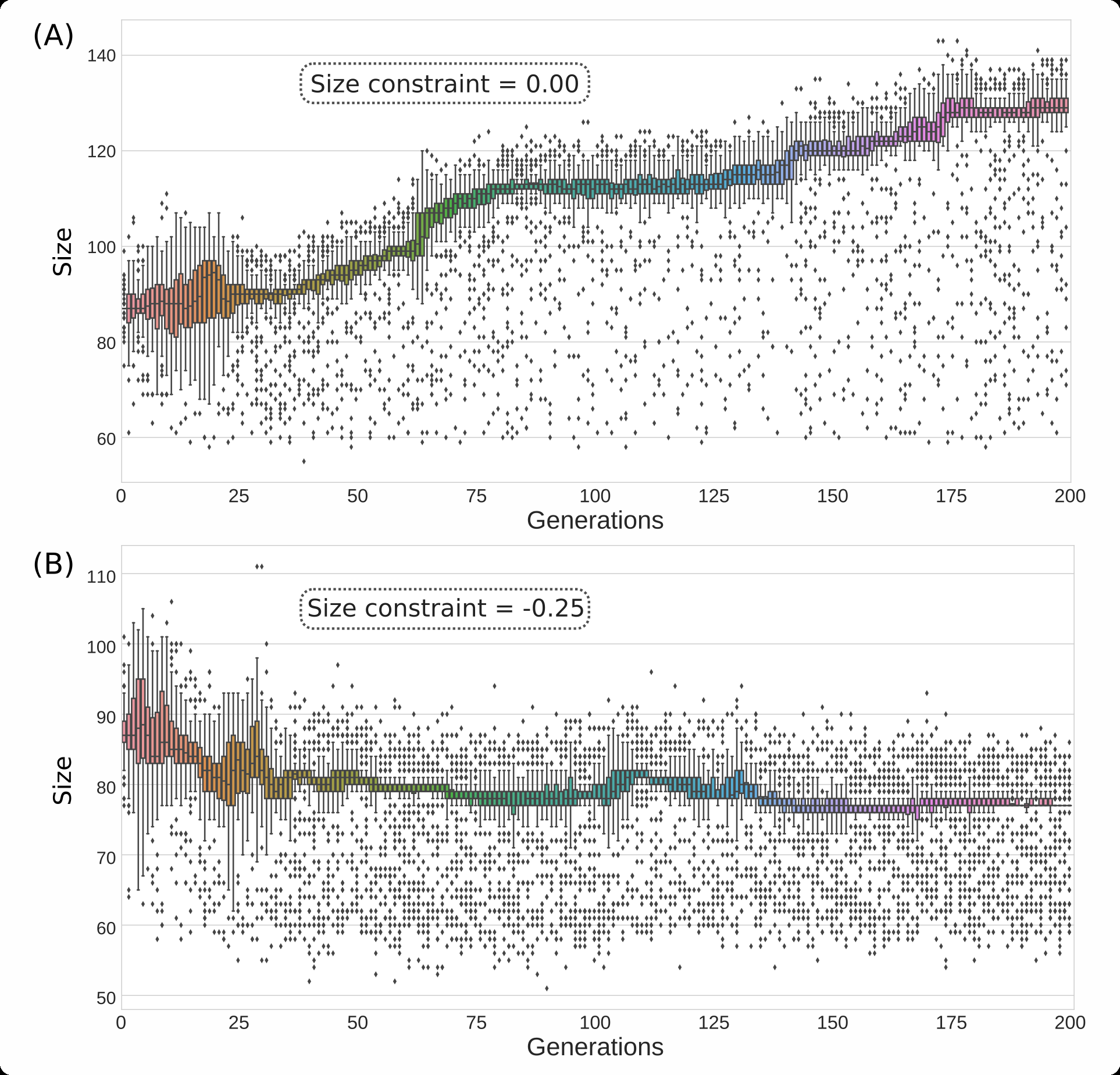

Supplement: S4 Fig — Number of metabolites contained in each individual (Y-axis) present in every generation over a typical evolution. The size constraint is a second weight applied on the fitness function. Here a weight of 1.0 is applied to maximize the MCC while a weight of 0.0 (A) and -0.25 (B) is applied to evolutions performed for 200 generations. In both cases, the individuals are initialized with a number of metabolites randomly varying from 60 to 100. Without applying a size constraint (A) the size of the individuals is ever increasing through the evolution. The application of a weight of -0.25 penalizes the individuals for the addition of metabolites and the individual size is stabilized (B). (TIF) [file pcbi.1006971.s004.tif]

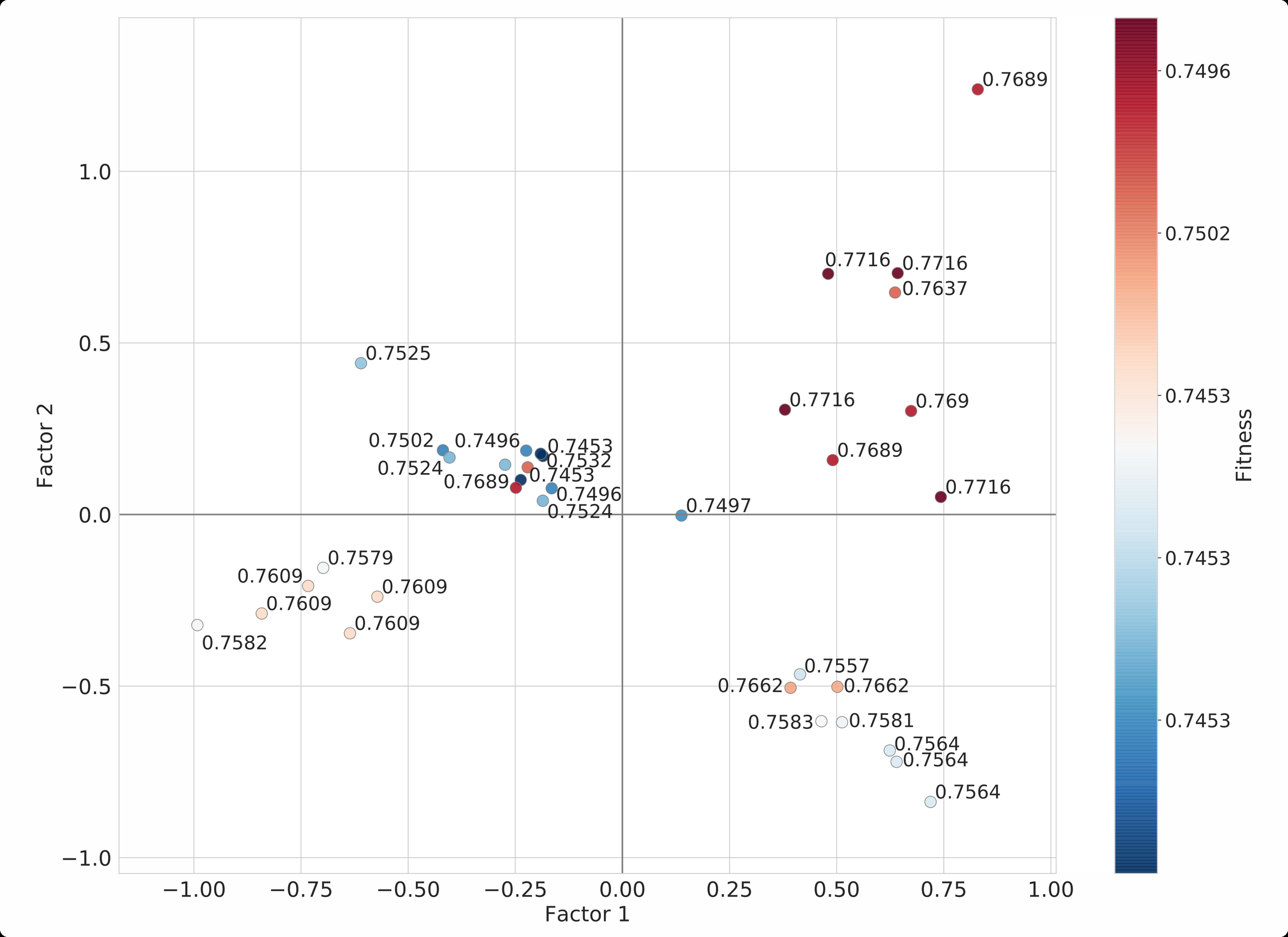

Supplement: S5 Fig — MCA is used to compare feature similarity between 35 individuals selected from HOFs from different evolutions. The fitness value is represented from the minimum value recorded (0.7453, blue) to the maximum (0.7716, red). The percent variability explained by the first two factors is 19.54% for factor 1 and 13.92% for factor 2. This analysis shows that similar fitness values can be obtained from diverse sets of metabolites. (TIF) [file pcbi.1006971.s005.tif]

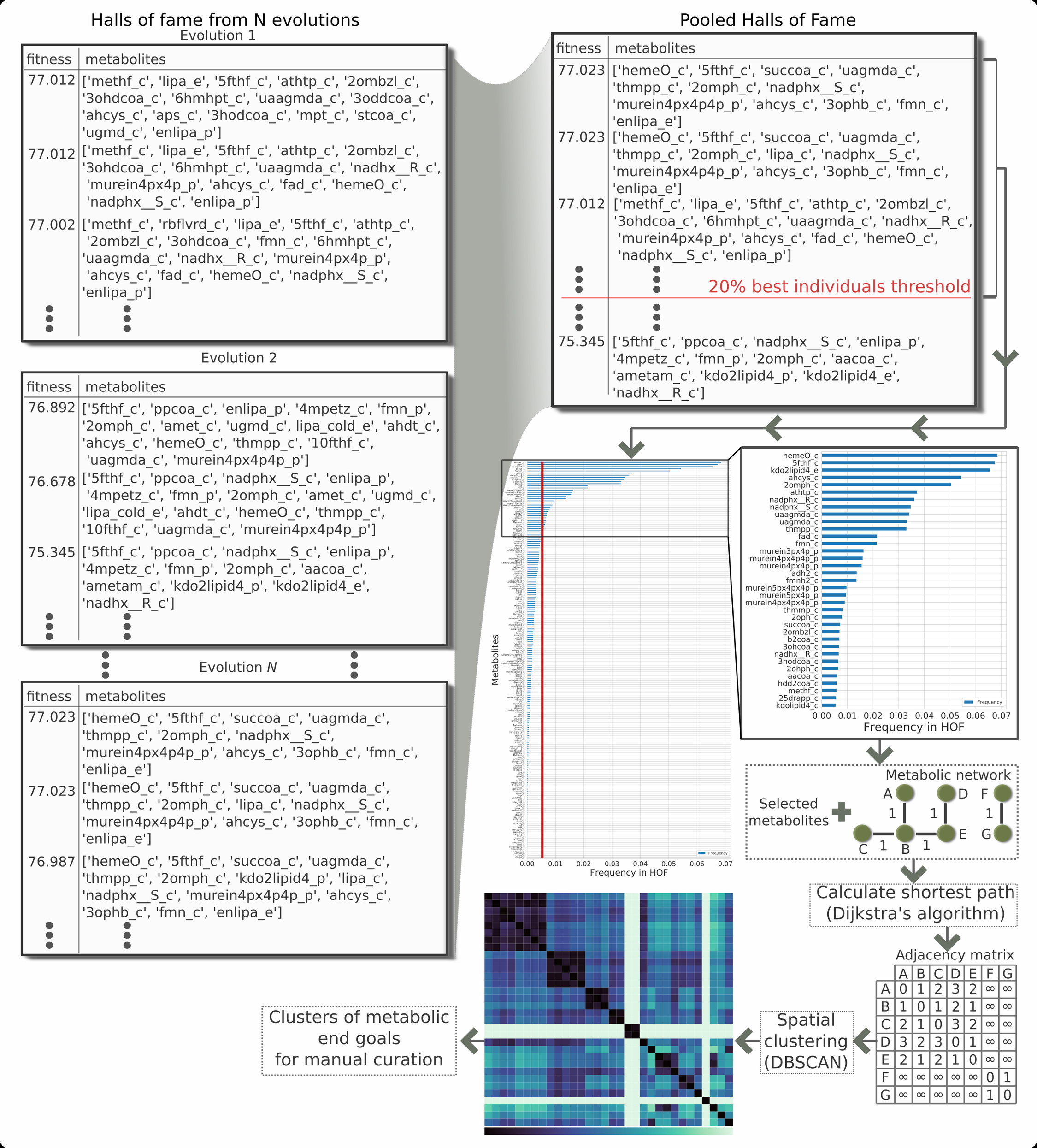

Supplement: S6 Fig — Each evolution generates a Hall of Fame (HOF) that, by default, stores the 1000 best individuals generated in the course of that evolution. The individuals produced by all the evolutions are pooled together and, arbitrarily, the 20% best individuals are selected based on their fitness value. The metabolites that compose these individuals are then ranked based on their frequency of apparition in those individuals. The metabolites with a frequency of apparition above average are selected. The Dijkstra algorithm is then used to calculate the distance in the metabolic network between the selected metabolites, assuming that a single reaction is one unit of distance. The distance matrix is then clustered using DBSCAN to identify clusters of metabolic end goals. The removal of highly connected metabolites may break the connection of certain metabolites to the rest of the network. For visualization purposes, the maximum distance is attributed to such metabolites. (TIF) [file pcbi.1006971.s006.tif]

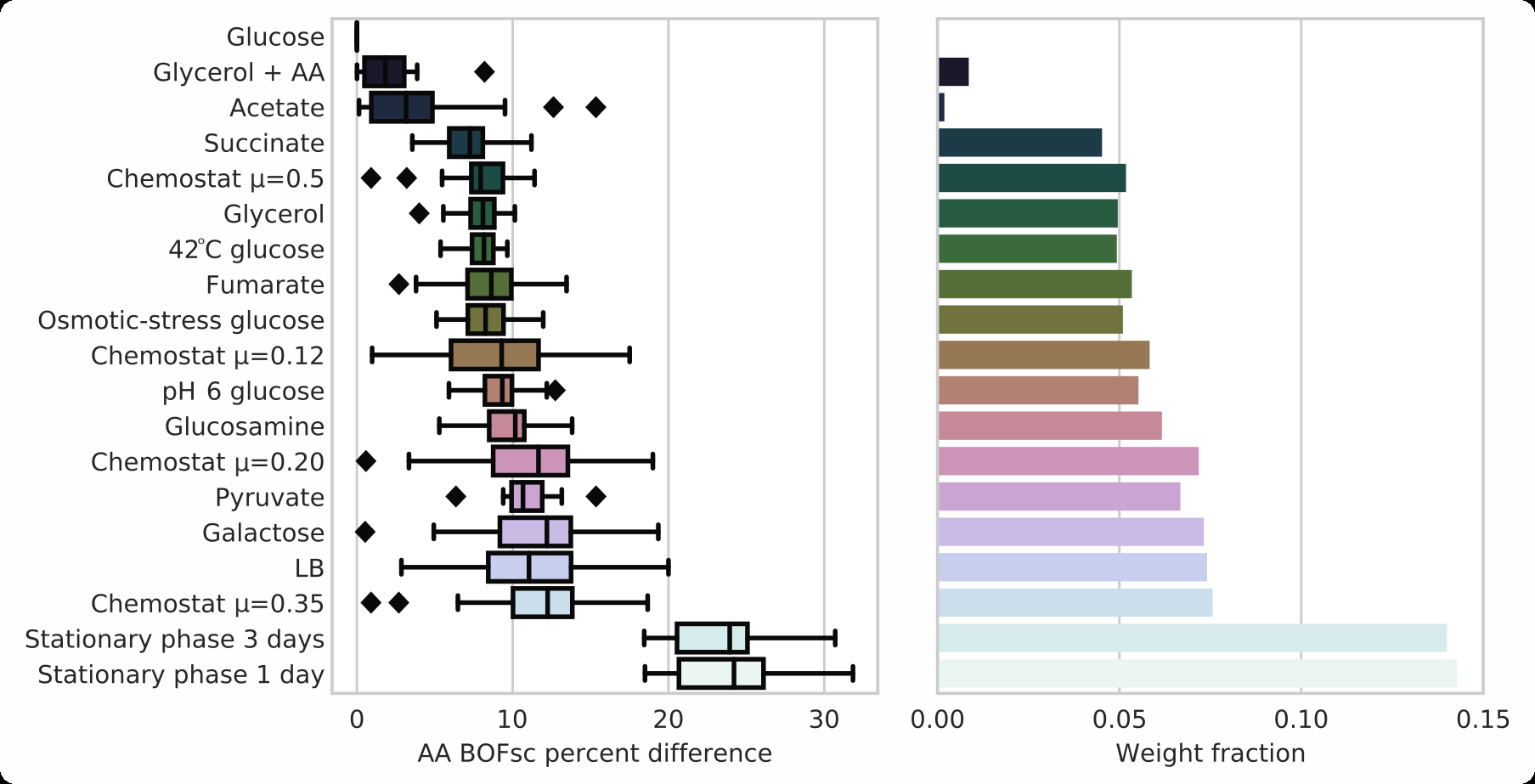

Supplement: S7 Fig — A high-quality quantitative proteomic dataset was used to generate stoichiometric coefficients for 18 different growth conditions [35]. Each boxplot represents the BOFsc percent difference of each amino acid (AA) in a given condition compare to glucose (left). The dataset also includes the macromolecular weight fraction (MWF) of protein in the cell for each condition. The histograms represent the difference between the protein weight fraction of each condition from the same dataset, against the fraction determined in the glucose growth condition (right). This shows that the stoichiometric coefficients are mainly affected by the MWF. (TIF) [file pcbi.1006971.s007.tif]

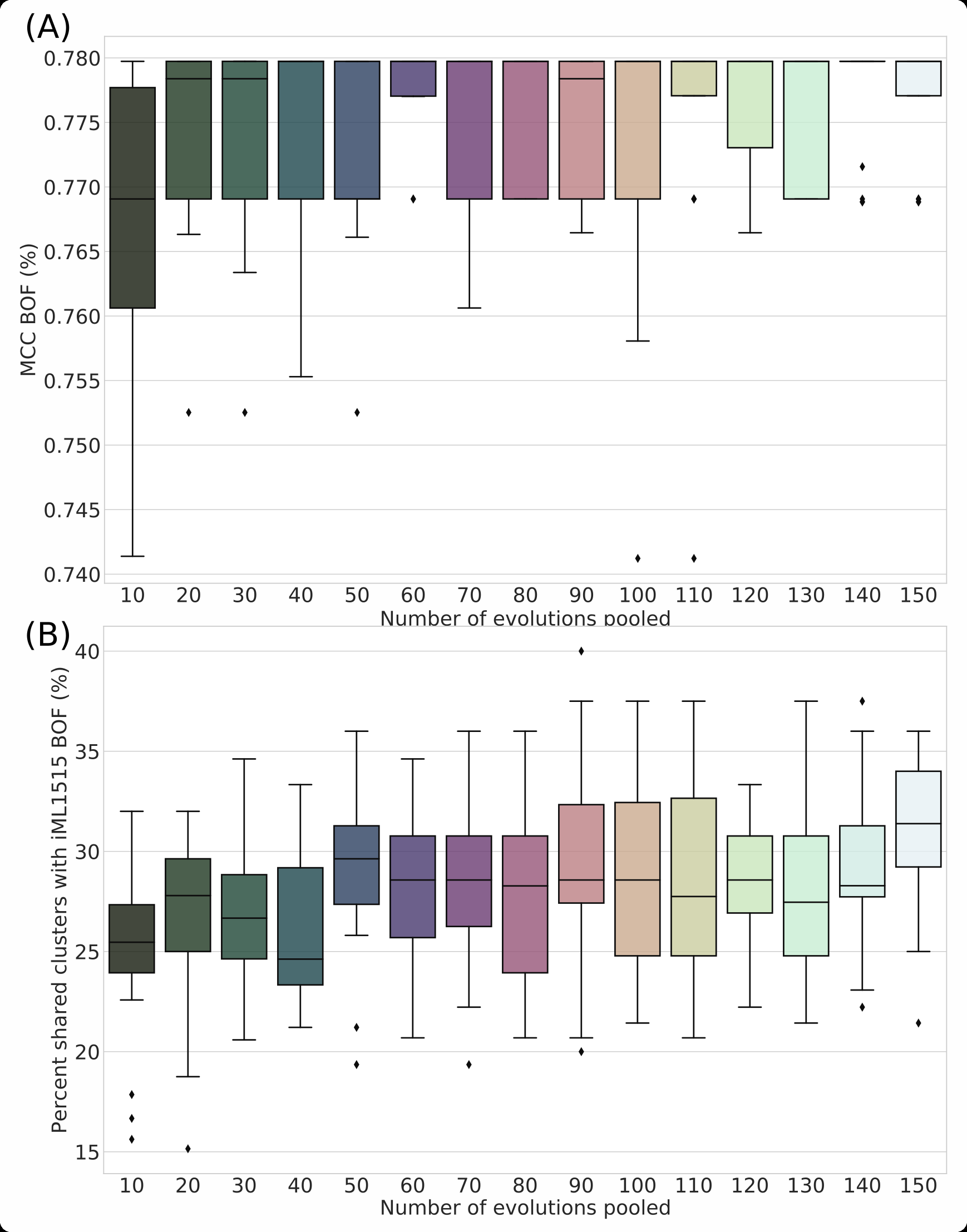

Supplement: S8 Fig — HOFs from the 150 evolutions over 500 generations are sampled, such that 20 samples are formed per number of evolutions pooled together (x-axis). The number of evolutions is the number of HOF pooled together to form clusters and select metabolites. (A) The MCC value of the automatic selection of metabolites following the clustering in BOFdat Step 3 for an increasing number of HOF from different evolutions. The median MCC value exceeds the baseline set by iML1515 for 20 evolutions pooled together and reaches a maximum of 0.779 after 40 evolutions. (B) The selected metabolites are compared against the original biomass of iML1515. New clusters are formed and the distribution of percentage of shared clusters is shown (y-axis). The use of more evolution yields an increase in the percentage of shared clusters with the original BOF from 26.61% for 10 evolutions to 31.19% at 150 evolutions. (TIF) [file pcbi.1006971.s008.tif]

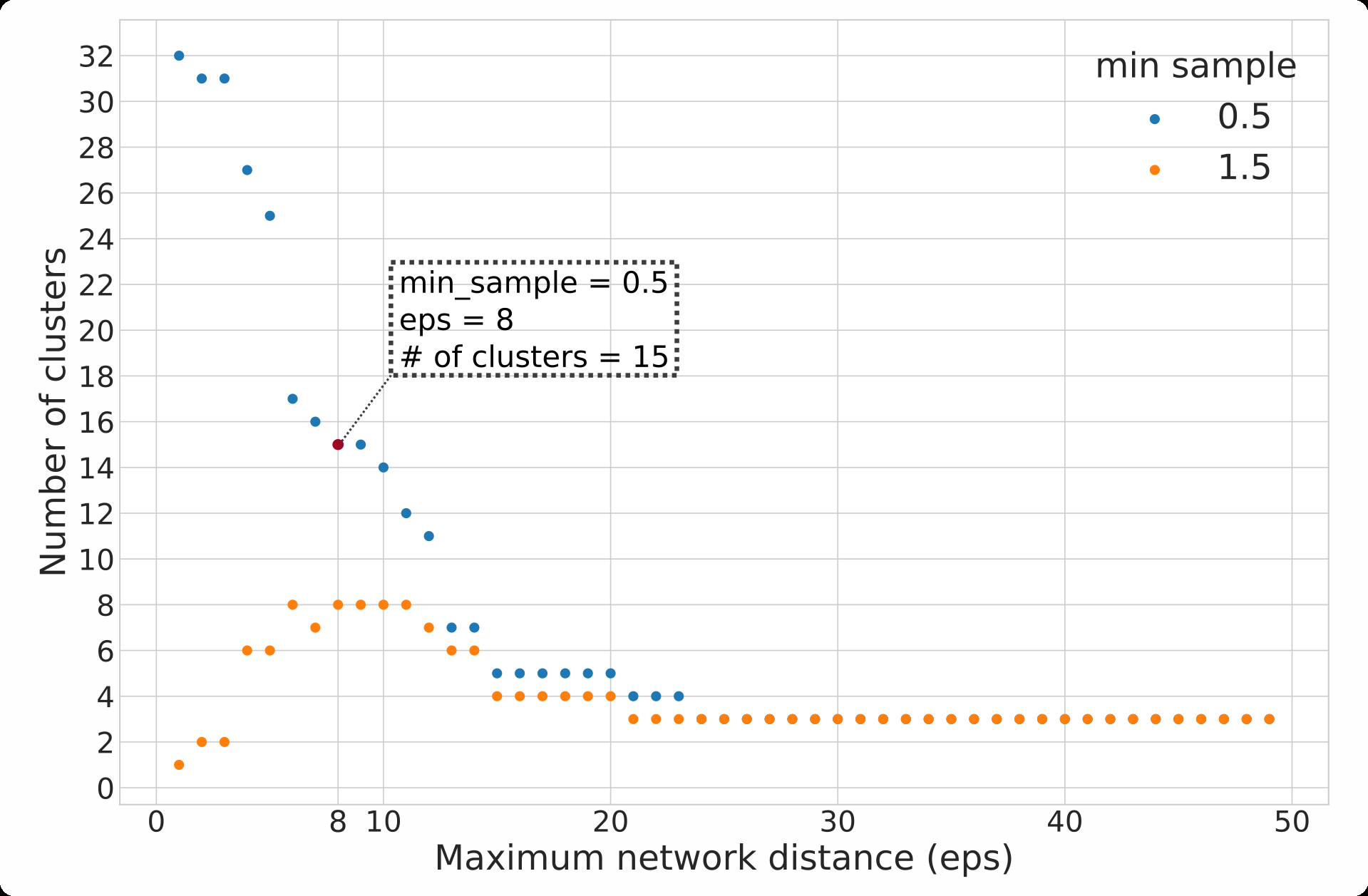

Supplement: S9 Fig — Selected metabolites from the 20% best individuals generated over 150 generations were clustered using the DBSCAN algorithm while varying the hyperparameters (eps and min_sample). As shown in the legend, two different values of min_sample were used (0.5 in blue, and 1.5 in orange). For these two min_sample values, the eps was varied from 1 to 50 and the number of clusters generated for these hyperparameters recorded (y-axis). Varying the “min_sample” for values 0 to 1 generated a single curve, while using a min_sample above 1 generated another. Hence only two values were represented. In this study, we systematically used a min_sample value inferior or equal to 1 (blue curve) and eps values that yielded a number of clusters around half of the number of metabolites provided. (TIF) [file pcbi.1006971.s009.tif]
