# Supplementary material for: BOFdat: Generating biomass objective functions for genome-scale metabolic models from experimental data
Source: PLoS Comput Biol. 2019 Apr 22;15(4):e1006971. doi: 10.1371/journal.pcbi.1006971 (PMC6497307; doi:10.1371/journal.pcbi.1006971)
Supplement: S1 Text — (PDF) [file pcbi.1006971.s012.pdf]

Supplementary Text

Implementation details

BOFdat Step 1

Generating stoichiometric coefficients from omic datasets and macromolecular weight fractions

The implementation of BOFdat is modular meaning that each function can be operated individually depending on the availability of data. Each module of BOFdat Step 1 calculates the stoichiometric coefficients for a different category of macromolecule. DNA, RNA, proteins and lipids stoichiometric coefficients can be calculated. To obtain the coefficients BOFdat requires the input of the macromolecular weight fraction (MWF) of the intended category. The MWF represents the total weight of a category of macromolecule within the cell dry weight. For example if 5% of the cell is composed of DNA, the DNA_{MWF} is 0.05 and stipulates that, in a steady-state growth scenario, for 1g of cell produced 0.05g of DNA is produced. To obtain a growth rate prediction, flux-balance analysis (FBA) relies on the definition of a basis where the product of cell mass by time is equal to 1g of dry weight per hour [1]. Using BOFdat and ensuring that all MWF used through the process add up to one ensures that this basis is respected and that the formulated growth rate prediction is valid.

DNA

The calculation of stoichiometric coefficients is made by counting the number of each bases in the provided fasta genome file such that:

$$R_{AT} = (A_f + T_f) / (L * 2) \qquad (1.1)$$

$$R_{CG} = (C_f + G_f) / (L * 2) \qquad (1.2)$$

where R_{AT} and R_{CG} are the ratios of the bases A, T and C, G, L is the length of the genome, A_f, T_f, C_f, G_f are the number of A, T, C and G bases counted in the single-strand genome sequence. Equation 2 is used to calculate the stoichiometric coefficient of each metabolite:

$$m_{sc} = R_m * DNA_{MWF} / (m_{MW} - Pi_{MW}) * 1000 \qquad (2)$$

where m_{sc} is the stoichiometric coefficient of a given base ($mmol * gDW$), R_m is the ratio of the metabolite for which the coefficient is determined, let it be R_{AT} or R_{CG}, within its category,

DNA_{MWF} is the MWF of DNA, m_{MW} is the molar weight of the metabolite, Pi_{MW} the molar weight inorganic di-phosphate (removed in the polymerization reaction).

RNA

The calculation of stoichiometric coefficients is made by counting the number of each bases in every gene in the provided GenBank annotation file such that:

(3) $$r_b = \left(\sum_{s_i}^{s_L} [s = b] \right) / l$$

where r_b is the ratio of base *b* in a gene of sequence length *l*. The ratio for each gene is then normalized by the abundance of each gene provided in the transcriptomic file (2 column CSV file where the first column represents the gene identifier and the second is the relative abundance of the transcript). The RNA content of the cell is divided in the three main categories present in the cell: rRNA, tRNA and mRNA. Since transcriptomic experiments usually deplete the rRNA and tRNA contents, the abundance of each rRNA or tRNA coding genes is considered equal to 1. Equation 4.1 allows to calculate the ratio of a given base in mRNA. Equation 4.2 is the simplification of equation 4.1 for the ratio of a given base in rRNA and tRNA, assuming each rRNA and tRNA have an abundance of 1.

(4.1) $$R_m = \sum_g (r_g * a_g) / \sum_g a_g$$

(4.2) $$R_r = R_t = \sum_g r_g / N$$

where R_m, R_r, R_t are the total fraction of the base *b* in mRNA, rRNA and tRNA respectively, r_g is the ratio of base *b* in gene *g* obtained in (3), a_g is the abundance of gene *g* . The total fraction of each base is then calculated by adding the content in each RNA category:

(5) $$R_b = (mRNA_f * R_m) + (tRNA_f * R_t) + (rRNA_f * R_r)$$

where $mRNA_f$, $tRNA_f$, $rRNA_f$ are the fraction of mRNA, tRNA and rRNA in the total RNA content of the cell. These values are set by default to $rRNA_f$ = 0.9, $tRNA_f$ = 0.05 and $mRNA_f$ = 0.05. The stoichiometric coefficient of a given base can then be determined:

(6) $$m_{sc} = R_b * RNA_{MWF} / (m_{MW} - Pi_{MW}) * 1000$$

where m_{sc} is the stoichiometric coefficient of a given base ($mmol * gDW$), R_b is the total ratio a given base in the entire RNA content of the cell obtained in (5), RNA_{MWF} is the total MWF of

RNA, m_{MW} is the molar weight of base *b* and Pi_{MW} is the molar weight of inorganic diphosphate released as a product of the polymerization reaction.

Protein

The calculation of stoichiometric coefficients for amino acids is based on the proteomic dataset and the MWF of proteins:

(7) $$r_a = \left(\sum_{s_i}^{s_L} [s = a] \right) / l$$

where r_a is the ratio of amino acid *a* in a protein of sequence length *l*. The ratio for each gene is then normalized by the abundance of each gene obtained by the proteomic dataset such that:

(8) $$R_a = \sum_p (r_a * a_p) / \sum_p a_p$$

where R_a is the total fraction of the amino acid *a*, r_a is the ratio of amino acid *a* for protein *p* obtained in (6), a_g is the abundance of gene *g* obtained via the proteomic data file (2 column CSV file where the first column represents the gene identifier and the second is the relative or absolute abundance of the transcribed protein). The total fraction of each amino acid can then be used to calculate their respective stoichiometric coefficient.

(9) $$m_{sc} = R_m * PROT_{MWF} / (m_{MW} - H_2O_{MW}) *1000$$

where m_{sc} is the stoichiometric coefficient of a given amino acid ($mmol * gDW$), R_b is the total ratio a given amino acid in the entire protein content of the cell obtained in (8), $PROT_{MWF}$ is the total MWF of proteins, m_{MW} is the molar weight of amino acid *a* and H_2O_{MW} is the molar weight of water released as a product of the polymerization reaction.

Lipids

The organism's lipid composition can be extracted through lipidomics experiments. Lipidomics allow to obtain the identity of the lipids that compose the cell membrane as well as their relative abundances. Unlike DNA, RNA and proteins that are composed of standard metabolites, the lipids may vary from a species to another and the mapping of the identifiers provided in the experimental data to model identifiers should be provided as a conversion file (2 column CSV file). The calculation of the lipid stoichiometric coefficients is then directly calculated from the relative abundances and the lipid MWF:

(10) $$m_{sc} = R_m * LIP_{MWF} / m_{MW} * 1000$$

where m_{sc} is the stoichiometric coefficient of a given lipid ($mmol * gDW$), R_m the relative abundance of a given lipid obtained from the lipidomic dataset, LIP_{MWF} the lipid MWF and m_{MW} the molar weight of lipid *m*.

Growth and non-growth associated maintenance

Maintenance costs represent the unspecified energy expenditure that are not directly part of the biomass precursor synthesis reactions. These costs lie outside of the scope of the metabolic network but are necessary to incorporate growth rate prediction in the model. The growth-associated maintenance (GAM) represents the ATP cost of generating another cell while the non-growth associated maintenance (NGAM) is associated with basal cellular processes. A standardized format for the organization of phenotypic data allows BOFdat to calculate GAM and NGAM (https://bofdat.readthedocs.io/).

Phenotypic data is parsed by BOFdat to constrain the provided model with the experimental growth rate, substrate uptake rate and the secretion rate of metabolic wastes. For a given experiment, a bound is set to the biomass objective function to set the growth rate to the experimental value. Similarly a flux is forced through the exchange reactions associated with either the uptake rate and the secretion rate(s). The model is then optimized for ATP production and the recorded flux through the ATP consumption is recorded. Going through all conditions allows to obtain a scatter plot where each growth rate is associated with an ATP cost. Linear regression is then applied and the slope of the curve is the GAM while the y-intercept is the NGAM.

BOFdat Step 2

Finding coenzymes

The degree of each metabolite is assessed by obtaining the number of reactions to which each metabolite takes part. The degree distribution for all metabolites in the *E. coli* metabolic network is shown in Fig S1. The threshold was set as the mean + one standard deviation for this distribution. Metabolites that were a result of BOFdat Step 1 and potentially included in the maintenance costs such as ATP, H+, ADP and PPi are removed from the list of potential metabolites to be added, but are still included in the calculation of statistic parameters of the distribution. One of the main reason to add this step is that metabolites with a high degree are likely to be missed by the genetic algorithm as many paths may lead to their production. Network topology nevertheless clearly informs on the importance of those metabolites.

Determining stoichiometric coefficients

The calculation of the stoichiometric coefficients is simplified for BOFdat Step 2 and Step 3 where the main focus is set on the qualitative choice of metabolites to add to the BOF. Hence, the assumption is that all metabolites within this category (i.e.: coenzymes and inorganic ions) are in equal abundance within the cell. The ratio of a given metabolite R_m is therefore:

$$R_m = 1 / N \quad (11)$$

where, N is the number of metabolites within the category. The stoichiometric coefficient m_{sc} of a given metabolite is then,

$$m_{sc} = R_m * CI_{MWF} / m_{MW} * 1000 \quad (12)$$

where CI_{MWF} is the molecular weight fraction of coenzymes and inorganic ions category and m_{MW} is molar weight of the metabolite.

BOFdat Step 3

Generation of initial populations

The initial population is generated by extracting the list of metabolites from the provided model in order to generate an index of metabolites for every individual in the population. If provided, the metabolites that belonged to the previous steps of BOFdat are removed from the list of all metabolites contained in the model. Then, each metabolite is tested individually for solvability by removing the existing objective function in the model and applying a single objective on the production of that given metabolite. A stoichiometric coefficient of 0.1 is attributed to the metabolite, making it a reactant. The reaction is a sink and has no product. The model is then optimized. A threshold for *in silico* growth rate value is set above the numerical error (1e-9) to determine whether or not the metabolite can be produced by the model. BOFdat does not change the media conditions of the model. Hence, modellers should provide the model with the exchange reactions set as the media used for the generation of the experimental data. Once a list of solvable metabolites is generated, a pre-feature selection step is executed that selects the metabolites that are most likely to affect the measure of fitness in the machine learning approach. Much like the selection of solvable metabolites this step iteratively sets each metabolite as an objective function. The difference here is that the gene essentiality is tested for each individual metabolite, allowing to attribute a Matthews Correlation Coefficient (MCC) value to each metabolite. The MCC is obtained using the SciKit Learn method (http://scikit-learn.org/). The metabolites are thus ranked in order of MCC and a threshold of mean + one standard deviation is set to determine those that should be used in the populations and the upcoming evolution (if a metabolite is absent from the initial population it will not be present in the evolution). Each individual is then generated randomly. The individual size is set to 20 meaning that 20 metabolites are identified as present in the population. The size of the population is adapted to get a sufficient coverage,

$$P = C * M/I \qquad (13)$$

where *P* is the population size, *C* is the coverage (set to 10 by default), *M* is the length of the index and *I* is the individual size. This adaptation of the population size to the number of features in the metabolite index is intended to reduce the initial population bias.For each individual the list of 0 and 1 defining the presence or absence of a metabolite is converted into an objective vector by applying a stoichiometric coefficient to the row of the stoichiometric matrix corresponding to a metabolite identified as present.

Implementation of the genetic algorithm

The DEAP toolbox is used to implement a genetic algorithm that maximizes the MCC between *in vitro* and *in silico* gene essentiality data. The initial population generated by the previous function is imported. Each column of the initial population is a different individual. Before the first

generation each individual is evaluated and ranked by fitness value. The fitness is parametrized by both MCC value (weight of 1.0) and by the size of the individual (weight of -0.25), which is the number of metabolites contained in the individual (Fig S4). This multi-parameter fitness function allows to select individuals with a minimal number of metabolites for a higher MCC value ensuring that selected metabolites are significant. The weights attributed to each parameter of the fitness function is one of the hyper-parameters that may be changed by the user. The individuals are selected with the tournament method (http://deap.readthedocs.io/). The mutation method applied is set to FlipBit (http://deap.readthedocs.io/) which is designed for binary individuals. The probability of selecting an individual to be mutated is set to 0.1 while the probability of flipping a feature is set to 0.005. The cross-over method applied is one point (http://deap.readthedocs.io/). This method randomly selects a location on the chromosome and exchanges both branches of the 2 individuals selected to breed together. The probability of crossover is set to 0.5. A common problem with genetic algorithms is the premature convergence to a local optima. The Random Offspring Generation (ROG) method is applied to avoid it [2]. Like other methods that avoid premature convergence, the ROG favors the generation of genetic diversity. To assess the loss of diversity, the offsprings produced by crossover are compared to their parents. If no difference is recorded between children and parents, identical twins were bred and the population did not gain diversity. One parent is then replaced by a completely new individual, generated in a random fashion as done for the initial population individuals and the crossover is re-applied. The algorithm selection, mutation and crossover process will run for a given number of generations as parametrized by the users.

Clustering into metabolic end goals

Each evolution generates a hall of fame (HOF) that contains the best individuals that were produced within it (http://deap.readthedocs.io). The default size of the HOF is 1000, meaning that the 1000 best individuals generated in an evolution are kept. To interpret the data generated by BOFdat over multiple evolutions, a spatial clustering method is implemented (Fig S6). The HOF of each evolutions are pooled together and an arbitrary threshold is applied to select the best individuals. By default, the 20% best are chosen. These selected individuals are then analyzed for their metabolite content. The frequency of apparition of each metabolite across all individuals is calculated and a threshold is set to keep only the most frequent, hereby reducing noise in data. The threshold is set to the mean of the frequency distribution, in the reconstruction of the *E.coli* biomass presented here (150 evolutions over 500 generations) the mean frequency was 0.00538. A distance matrix is generated using the Dijkstra algorithm finding the shortest path between nodes. Hubs are known to introduce bias in paths, greatly shortening paths between nodes that are not closely related. Therefore, nodes with more than 15 connections are removed from network distance analysis. This number may vary from a metabolic network to another depending on the number and degree of each metabolite, hence the related threshold may be set by users as described in the documentation (https://bofdat.readthedocs.io/). The adjacency matrix obtained for the selected metabolites is clustered using the DBSCAN [3] algorithm from the SciKit Learn library (http://scikit-learn.org/). Unlike hierarchical clustering methods, DBSCAN does not require *a priori* definition of the

number of clusters but requires the minimum number of elements to include in a cluster, (suggested and default value of 0.5), and the maximum distance between elements to form a cluster, termed eps in the DBSCAN documentation. This second value greatly impacts the cluster formation as a greater eps would group all metabolites into a single cluster (i.e.: 30) whereas a shorter eps (i.e.: 1) separates every metabolite into a unique cluster. The impact of varying the eps is studied in Fig S9. As mentioned, when a low eps is given, the number of clusters is maximized to a point where the number of clusters is equivalent to the number of metabolites, providing no mechanistic explanation whereas a greater eps yields very few clusters, also making the interpretation difficult. For this study we have used eps values that yield a number of clusters at half the number of provided metabolites and have come with results that could be interpreted by a modeller with knowledge of the organism.

BOFdat provides a way to automatically select metabolites from the formed clusters. The cluster frequency is calculated by adding metabolite frequency:

(14) $$C_F = \sum_{m_i}^{i} m_F$$

The z-score of each cluster is than calculated:

(15) $$C_z = \frac{C_F - \overline{x_C}}{S_C}$$

where C_z is the z-score of a cluster, $\overline{x_C}$ the average of all cluster frequencies, S_C the standard deviation of all cluster frequencies and C_F the individual cluster frequency obtained from (1). Each cluster is then weighted according to its z-score:

(16) $$C_z \geq 1,\ w_C = 3\,,$$
$$1 > C_z \geq 0,\ w_C = 2\,,$$
$$C_z < 0,\ w_C = 1\,.$$

The weight of each cluster determines the number of metabolites that should be added to the final BOF suggested by BOFdat. If the weight is superior to the number of metabolites in the cluster, the number of metabolites added is equal to the size of the cluster. The stoichiometric coefficients of each metabolite added in BOFdat Step 3 are determined in a similar way as for BOFdat Step 2 where the ratio of each metabolite is by default distributed equally across all metabolites present in that step (Eq. 11 and 12).

Methods

Using omic datasets to calculate stoichiometric coefficients

BOFdat uses both omic datasets and macromolecular weight fractions (MWF) to calculate stoichiometric coefficients. The omic datasets allow to get the relative abundance of transcribed proteins or RNA transcripts. To evaluate the importance of these parameters, we calculated stoichiometric coefficients for amino acids under 18 different experimental conditions. A high-quality proteomic dataset for *E.coli* was generated by Heinemann and colleagues [4] under multiple experimental conditions. Along with the precise quantity of each protein present in the cell, the protein weight fractions were also measured for every growth conditions making the data perfectly suitable for our study. Using equation 17, stoichiometric coefficients for all growth conditions were compared to the data obtained on glucose minimal media (Fig S7):

$$e_a = \frac{|n_a - g_a|}{g_a} * 100 \qquad (17)$$

where e_a is the percentage difference between the reference coefficient and the new coefficient for a given amino acid, n_a is the new coefficient in the given experimental condition, g_a is the original coefficient on glucose minimal media. When using condition-specific protein weight fractions, the mean difference from glucose was between 4.1% (Acetate) and 24.2% (Stationary phase 1 day). The weight fraction differences correlated with the average percent difference for each condition (Pearson *r* = 0.984, *p-value* = 3.610e-14) indicating that the weight fraction is the most prevailing parameter to determine stoichiometric coefficients. The use of appropriate weight fractions has a greater impact on the stoichiometric coefficients generated and should be considered as the most significant experimental measurement in the biomass composition of GEMs.

Required number of evolutions

To provide a recommendation for the number of evolutions that should be performed by a user of BOFdat we generated the MCC values and the number of shared clusters with *i*ML1515 wild-type BOF for a number of evolutions pooled together ranging from 10 to 150 pooled evolutions (Fig S8). 20 random samples were formed for each number of pooled evolutions. For each sample the evolutions were randomly selected from 187 individual evolutions. A selection of metabolites was operated as described above for each sample generated. The metabolite selection was then used to evaluate the MCC value of the given solution (Fig S8A). Similarly, for each sample, the selected metabolites were pooled with the metabolites from the original *i*ML1515 BOF and clustered using the method described above (eps = 8, min_sample = 0.5) (Fig S8B). The maximum MCC value obtained was 0.779 and was observed as the median for more than 40 evolutions pooled together. For users with fewer computational resources, we note that pooling 20 evolutions had a median MCC higher than the baseline established by

*i*ML1515 at 0.775. The percent shared clusters with *i*ML1515 steadily increased between 10 evolutions (26.61%) and 150 evolutions (31.19%), meaning that the accuracy of the metabolites found by BOFdat is dependent to the number of evolutions generated.

Multiple correspondence analysis

Multiple correspondence analysis (MCA) was used to compare feature selected by different evolutions [5]. The presence or absence of a metabolite in an individual is defined as a nominal variable with two levels (present = 1, absent = 0) and the total number of nominal variables K is the number of unique metabolites in all selected individuals (119 metabolites). The number of observations I is defined as the total number of individuals compared to each other. We selected 35 individuals with fitness values ranging from the lowest to the highest recorded in the 150 evolutions used in this study. The 35 individuals were extracted from 10 different HOFs. The X matrix of size K feature columns by I observations rows was processed following the usage guide from the Python MCA package (https://pypi.org/project/mca/).

Biomass objective function from SEED

The SEED platform for automated reconstruction of genome-scale models of metabolism allows for the selection of pre-defined biomass objective function that is based on knowledge (File S1). The default gram-negative bacteria BOF was chosen when reconstructing the model for *Escherichia coli* MG1655 on the SEED platform. The objective function was extracted from the model and the SEED metabolite identifiers were converted to BiGG identifiers using MetaNetX [6]. The remaining unconverted metabolites were converted manually to the best matching BiGG identifier.

Using BOSS on a genome scale

We used the BOSS (Biological Objective Solution Search) method [7] to generate a biomass objective reaction from fluxomics data. We used ^{13}C metabolic flux analysis data for *E. coli* grown on glucose minimal medium [8]. To solve BOSS for the genome-scale model [9], we used a recent algorithmic extension of BOSS, which solves the nonconvex optimization problem of BOSS using a distributed optimization method called ADMM (alternating direction method of multipliers) [10]. We made three additional modifications to the extended BOSS for this study. First, we constrained BOSS to keep the biomass coefficients from BOFdat Step 2, to facilitate the comparison between BOFdat Step 3 vs. BOSS. Second, because ADMM can require many iterations to reach a high-precision solution, we implemented a solution "polishing" step. The polishing step enables taking a medium-precision solution from ADMM (e.g., feasibility tolerance ~ 10^{-6}) and subsequently solving one optimization problem to obtain a high-precision solution (e.g., feasibility tolerance ~ 10^{-9}). The polishing step solves the following quadratic program (QP):

(18) $$\min_{v,y} \|y - y^B\|^2{}_2 \text{ subject to } Sv + yz^B = 0,\ l \le v \le u$$

where v is the vector of fluxes, y is the vector of stoichiometric coefficients for the generated objective, y^B is the stoichiometric vector from the BOSS solution, z^B is the flux through the BOSS objective reaction, S is the stoichiometric matrix for the metabolic reconstruction, and l and u are lower and upper flux bounds, respectively. The solution to this problem provides the objective reaction that most closely matches the BOSS solution that still satisfies the FBA constraints to a higher precision. Third, we generated a sparse objective using both an L1-norm regularization during the BOSS computation, and also a post-processing step. For post-processing, we sparsified the vector, y^B, in the polishing step (QP) above by only keeping the largest 200 coefficients ordered by magnitude and zeroing out the rest of the coefficients. Therefore, the solution to the polishing step generated a sparsified objective that still satisfies FBA constraints.

Levenshtein distance calculation

The Levenshtein distance allows to compute distance between strings of different lengths. The BOF generated by all three steps of BOFdat, SEED, BOSS and the biomass of a completely different organism (yeast, *i*MM904) were compared to the original *i*ML1515 wild-type biomass using this distance metric. The list of metabolites from the reference (original *i*ML1515) and the compared biomass are converted to strings where each letter corresponds to the presence or absence of a metabolite in the reference. The reference as a presence indicator for each metabolite whereas the compared biomass as both presence or absence. In that case, the Levenshtein distance is equivalent to the number of absence indicators.

References

1. Varma A, Boesch BW, Palsson BO. Stoichiometric interpretation of Escherichia coli glucose catabolism under various oxygenation rates. Appl Environ Microbiol. 1993;59: 2465–2473. Available: https://www.ncbi.nlm.nih.gov/pubmed/8368835

2. Rocha M, Neves J. Preventing Premature Convergence to Local Optima in Genetic Algorithms via Random Offspring Generation. Multiple Approaches to Intelligent Systems. Springer Berlin Heidelberg; 1999. pp. 127–136. doi:10.1007/978-3-540-48765-4_16

3. Ester M, Kriegel H-P, Sander J, Xu X, Others. A density-based algorithm for discovering clusters in large spatial databases with noise. Kdd. 1996. pp. 226–231. Available: http://www.aaai.org/Papers/KDD/1996/KDD96-037.pdf

4. Schmidt A, Kochanowski K, Vedelaar S, Ahrné E, Volkmer B, Callipo L, et al. The quantitative and condition-dependent Escherichia coli proteome. Nat Biotechnol. 2016;34: 104–110. doi:10.1038/nbt.3418

5. Abdi H, Valentin D. Multiple correspondence analysis. Encyclopedia of measurement and statistics. 2007; 651–657. Available: https://www.researchgate.net/profile/Dominique_Valentin/publication/239542271_Multiple_Correspondence_Analysis/links/54a979900cf256bf8bb95c95.pdf

6. Moretti S, Martin O, Van Du Tran T, Bridge A, Morgat A, Pagni M. MetaNetX/MNXref--reconciliation of metabolites and biochemical reactions to bring together genome-scale metabolic networks. Nucleic Acids Res. 2016;44: D523–6. doi:10.1093/nar/gkv1117

7. Gianchandani EP, Oberhardt MA, Burgard AP, Maranas CD, Papin JA. Predicting biological system objectives de novo from internal state measurements. BMC Bioinformatics. 2008;9: 43. doi:10.1186/1471-2105-9-43

8. Van Rijsewijk BRBH, Nanchen A, Nallet S, Kleijn RJ, Sauer U. Large-scale 13C-flux analysis reveals distinct transcriptional control of respiratory and fermentative metabolism in Escherichia coli. Mol Syst Biol. EMBO Press; 2011;7: 477. Available: http://msb.embopress.org/content/7/1/477.short

9. Monk JM, Lloyd CJ, Brunk E, Mih N, Sastry A, King Z, et al. iML1515, a knowledgebase that computes Escherichia coli traits. Nat Biotechnol. 2017;35: 904–908. doi:10.1038/nbt.3956

10. Yang L, Bento J, Lachance J-C, Palsson BO. Genome-scale estimation of cellular objectives [Internet]. arXiv [q-bio.QM]. 2018. Available: http://arxiv.org/abs/1807.04245
